# Ictal wavefront propagation in slices and simulations with conductance-based refractory density model

**DOI:** 10.1371/journal.pcbi.1009782

**Published:** 2022-01-18

**Authors:** Anton V. Chizhov, Dmitry V. Amakhin, Elena Yu. Smirnova, Aleksey V. Zaitsev

**Affiliations:** 1 Laboratory of Molecular Mechanisms of Neural Interactions, Sechenov Institute of Evolutionary Physiology and Biochemistry of the Russian Academy of Sciences, Saint Petersburg, Russia; 2 Computational Physics Laboratory, Ioffe Institute, Saint Petersburg, Russia; 3 Institute of Experimental Medicine, Almazov National Medical Research Centre, Saint Petersburg, Russia; University of California San Diego, UNITED STATES

## Abstract

The mechanisms determining ictal discharge (ID) propagation are still not clear. In the present study, we aimed to examine these mechanisms in animal and mathematical models of epileptiform activity. Using double-patch and extracellular potassium ion concentration recordings in rat hippocampal-cortical slices, we observed that IDs moved at a speed of about 1 mm/s or less. The mechanisms of such slow propagation have been studied with a mathematical, conductance-based refractory density (CBRD) model that describes the GABA- and glutamatergic neuronal populations’ interactions and ion dynamics in brain tissue. The modeling study reveals two main factors triggerring IDs: (i) increased interneuronal activity leading to chloride ion accumulation and a consequent depolarizing GABAergic effect and (ii) the elevation of extracellular potassium ion concentration. The local synaptic transmission followed by local potassium ion extrusion and GABA receptor-mediated chloride ion accumulation underlies the ID wavefront’s propagation. In contrast, potassium ion diffusion in the extracellular space is slower and does not affect ID’s speed. The short discharges, constituting the ID, propagate much faster than the ID front. The accumulation of sodium ions inside neurons due to their hyperactivity and glutamatergic currents boosts the Na^+^/K^+^ pump, which terminates the ID. Knowledge of the mechanism of ID generation and propagation contributes to the development of new treatments against epilepsy.

## Author summary

During an epileptic seizure, neuronal excitation spreads across the brain tissue and is accompanied by significant changes in ionic concentrations. Ictal discharge front spreads at low speeds, less than 1 mm/s. Mechanisms underlying this phenomenon are not yet well understood. We study these mechanisms using electrophysiological recordings in brain slices and computer simulations. Our detailed biophysical model describing neuronal populations’ interaction, spatial propagation, and ionic dynamics reproduces the generation and propagation of spontaneously repeating ictal discharges. The simulations are consistent with our recordings of the electrical activity and the extracellular potassium ion concentration. We distinguished between the two alternative mechanisms of the ictal wavefront propagation: (i) the diffusion of potassium ions released from excited neurons, which depolarizes distant neurons and thus supports excitation, and (ii) the axonal spread of excitation followed by the local extracellular potassium ion accumulation that supports the excitation. Our simulations provide evidence in favor of the latter mechanism. Our experiment-based modeling contributes to a mathematical description of brain tissue functioning and potentially contributes to developing new treatments against epilepsy.

## Introduction

The exact nature of spatiotemporal dynamics of brain activity during seizures is of great importance. However, the mechanisms of epileptic discharge propagation are not yet well understood. Epileptic activity in the brain is divided into several forms, including long-lasting (with a duration of tens of seconds) ictal discharges (IDs) and different types of short-lasting discharges (SDs) of less than one or a few seconds [1]. Several forms of SDs are distinguished: (i) predominantly, GABAergic interictal discharges (IIDs) emerging between IDs; (ii) IID-like preictal discharges (PIDs) that may take place just before an ID; and (iii) GABA-glutamatergic late short discharges (LSDs) that constitute either an ID in its late phase (known as intraictal bursts in [2]) or continuously repeating in status epilepticus. These discharges propagate through the brain tissue at different speeds. The typical speed of SD propagation is about tens of millimeters per second [2–6]. ID propagation is slower; the rate is less than 1 mm/s in the cerebral cortex of patients [7–9] and animals *in vivo* [10,11] and *in vitro* [2,3,12–14]. The difference in speeds of IDs and SDs suggests different mechanisms of propagation. The consensus among previous studies is that SD generation and propagation mechanisms are not related to ion dynamics, which implies only neuronal excitation and synaptic interactions [6,15–17]. In contrast, the generation of IDs critically depends on ion dynamics [18–21], which is often mistakenly omitted in many other modeling studies. Whether the contribution of the ion dynamics into the ID propagation is direct—due to ion diffusion—or indirect—as a consequence of neuronal excitation—is still debated [22].

A slowly propagating wave of ID is accompanied by a wave of increasing extracellular potassium ion concentration [1]. However, it is still unclear whether ictal activity causes an increase in potassium ion concentration or, conversely, diffusion of potassium ions from sites of high concentration causes ictal activity. Therefore, at least two alternative mechanisms of ID propagation have been suggested: (i) synaptic transmission between neurons, including spike propagation through axons and passive conduction of postsynaptic signals through dendritic neuronal branches [23,24]; (ii) the diffusion of potassium ions, resulting from substantial transient increases in extracellular potassium ion concentrations that are observed during IDs [1,25,26].

Recently, using the proposed spatially distributed extension of the simple model of IDs and SDs Epileptor-2 [27], we compared the impacts of synaptic transmission and potassium ion diffusion in 2-D simulations [28]. We showed that potassium ion diffusion can contribute to ictal discharge propagation; however, it leads to an ID propagation speed of much less than 0.1 mm/s. Instead, the synaptic connectivity-based mechanism provides a speed comparable to experimental data. However, due to the reductions made in the Epileptor-2, it cannot be compared to electrophysiological experiments in detail and, in particular, does not exclude any indirect effects of potassium ion diffusion. Therefore, here we use our detailed mathematical model based on the conductance-based refractory density (CBRD) approach and extend it to the 1-D spatially distributed case. We simulate the propagation of repeating IDs and compare the simulations with our recordings of neuronal electrical activity and extracellular potassium ion concentration in two points of the entorhinal cortex (EC) at a distance of 2 mm in rat brain slices in the 4-aminopyridine model. The obtained results indicate that the accumulation of extracellular potassium ions due to synaptic activity but not potassium ion diffusion promotes ID propagation.

## Results

### Experimental observations of ictal front propagation

**Fig 1:**
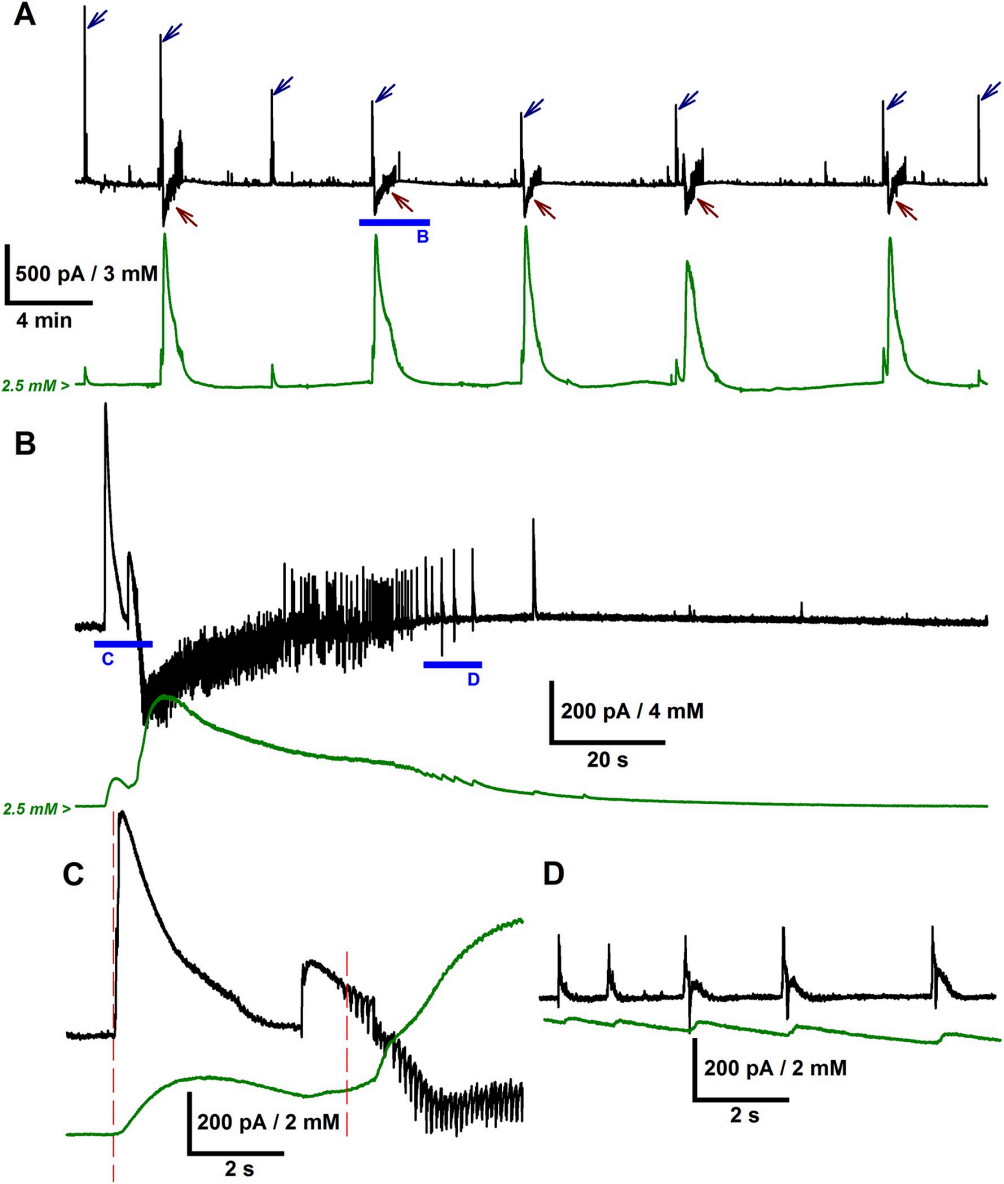
A neuronal and ionic dynamics correlation is revealed with the simultaneous whole-cell patch-clamp and extracellular potassium ion concentration recordings during epileptiform activity in rat ERC. (A) The representative recordings of synaptic currents in the voltage-clamp mode (black trace) and extracellular potassium ion concentration (green trace). *V*_hold_ = –27 mV, which is between the reversal potentials of GABA_A_ and glutamate receptor-mediated currents. Outward GABA-mediated synaptic current corresponds to IIDs and PIDs and is marked with blue arrows. Inward, predominantly glutamate-mediated currents correspond to IDs and are marked with brown arrows. Note that all IIDs, PIDs, and IDs induce K^+^ transients. The blue bar marks a fragment of the recording, which contains PID and ID and is extended in (B). (B) K^+^ transient has a two-stage rise, corresponding to PID and ID initiation, respectively. The ID initiation phase is extended in (C). (C) Red dashed lines indicate the start of the PID and the emergence of glutamate-mediated components at the beginning of the ID. The late stage of the ID consisted of short discharges, which are extended in (D). (D) Each of these late-stage discharges produces a transient increase of [K^+^]_o_.

We induced the epileptiform activity in rat brain slices and performed the simultaneous recordings of the synaptic currents and the [K^+^]_o_ fluctuations (Fig 1A). The epileptiform activity in the implemented *in vitro* model was described in detail in our previous works [21,29,30]. Briefly, two main types of epileptiform discharges were observed: GABA-mediated IIDs (marked with blue arrows in Fig 1A) and subsequent IDs (marked with brown arrows in Fig 1A), which are mediated by both GABA and glutamate. IIDs had a duration of about 1 s and, in some cases, transitioned to the IDs, which had the duration of 25–100 s and emerged every 5–10 min. The IIDs, which directly preceded IDs, will be referred to as preictal discharges (PIDs).

During each ID, we observed a substantial increase in extracellular potassium ion concentration (Fig 1B). In most cases, the rise in potassium ion concentration came in two stages (Fig 1C). The first minor rise of [K^+^]_o_ corresponded to the GABA-mediated PID. The second more significant rise of [K^+^]_o_ occurred during the tonic phase of ID and coincided with the emergence of glutamate-mediated currents.

[K^+^]_o_ peaked during the tonic phase of the ID (the average peak [K^+^]_o_ was 9.5 ± 0.3 mM, *n* = 27 IDs from nine slices), after which it gradually decreased. The late-stage clonic discharges produced transient increases of [K^+^]_o_ (Fig 1D), which did not change the overall decreasing trend. In half of the IDs included in the analysis, the K^+^ concentration transiently decreased below the level observed before the ID.

**Fig 2:**
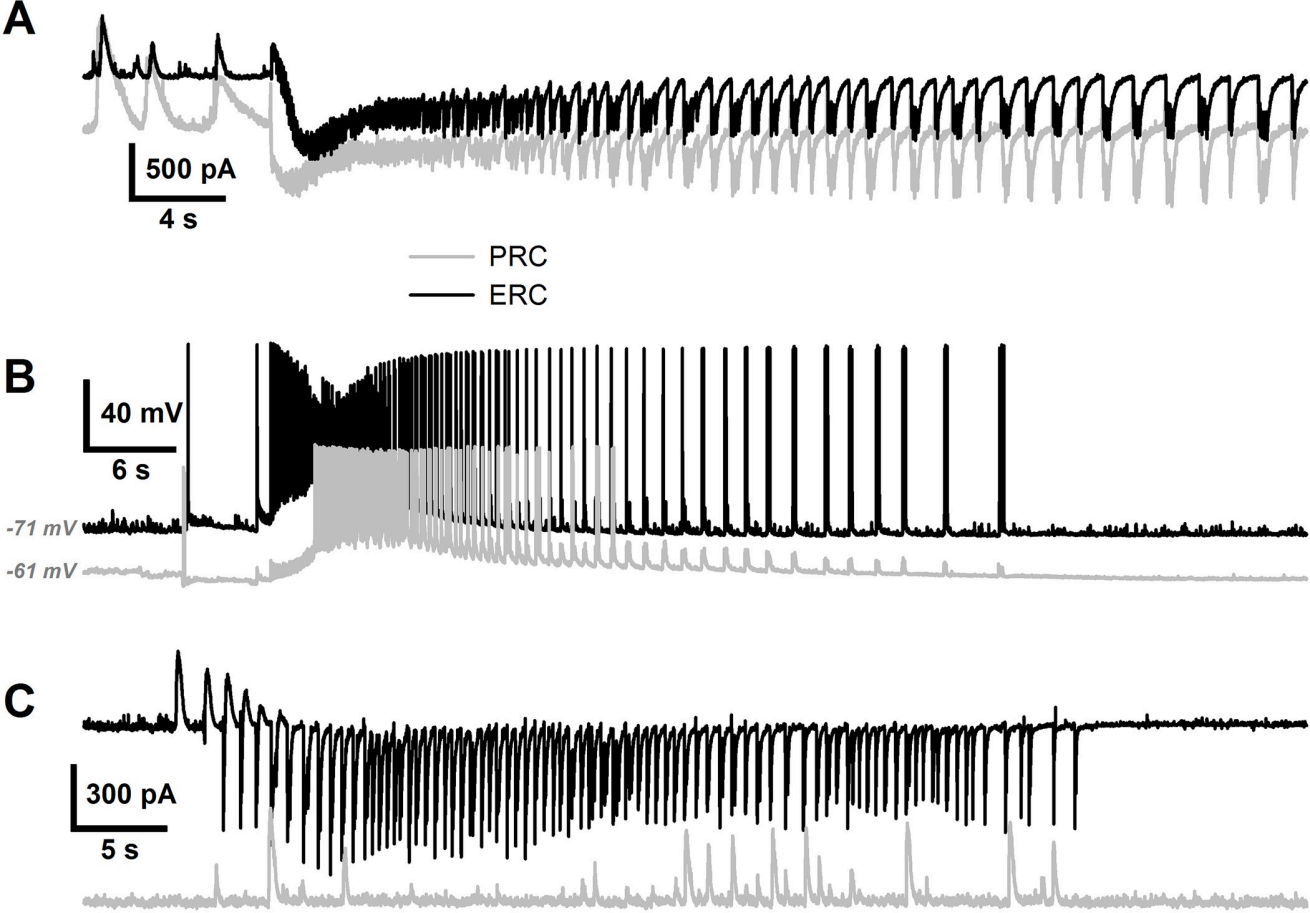
The representative simultaneous whole-cell patch-clamp recordings of IDs in the ERC and PRC. (A) Dual voltage-clamp recordings (*V*_hold_ = –27 mV) of synaptic activity during IDs at a distance of 2 mm. A delay between the tonic phases of the IDs is observed. (B) Dual current-clamp recordings of membrane voltage during IDs. Note the delay between the IDs, which is comparable to the one in A. (C) A representative dual voltage-clamp recording illustrating the absence of ID propagation from one cortical area to another in the slice.

Next, we investigated the ID propagation in the neocortex performing simultaneous whole-cell patch-clamp recordings in the entorhinal (ERC) and perirhinal cortices (PRC) at a distance of 2 mm between the recording electrodes. In 85% of cases included in the analysis (with the delays longer than 1 s), we detected the IDs in both cortical areas with a delay in the initiation of several seconds (Fig 2), thus obtaining a wide variety of speed values of the order of magnitude of tenths of a millimeter per second. The delay between the IDs was observed in both voltage-clamp and current-clamp modes (Fig 2A and 2B, respectively). Some IDs (15%) failed to propagate and could be detected in only one cortical area (Fig 2C).

**Fig 3:**
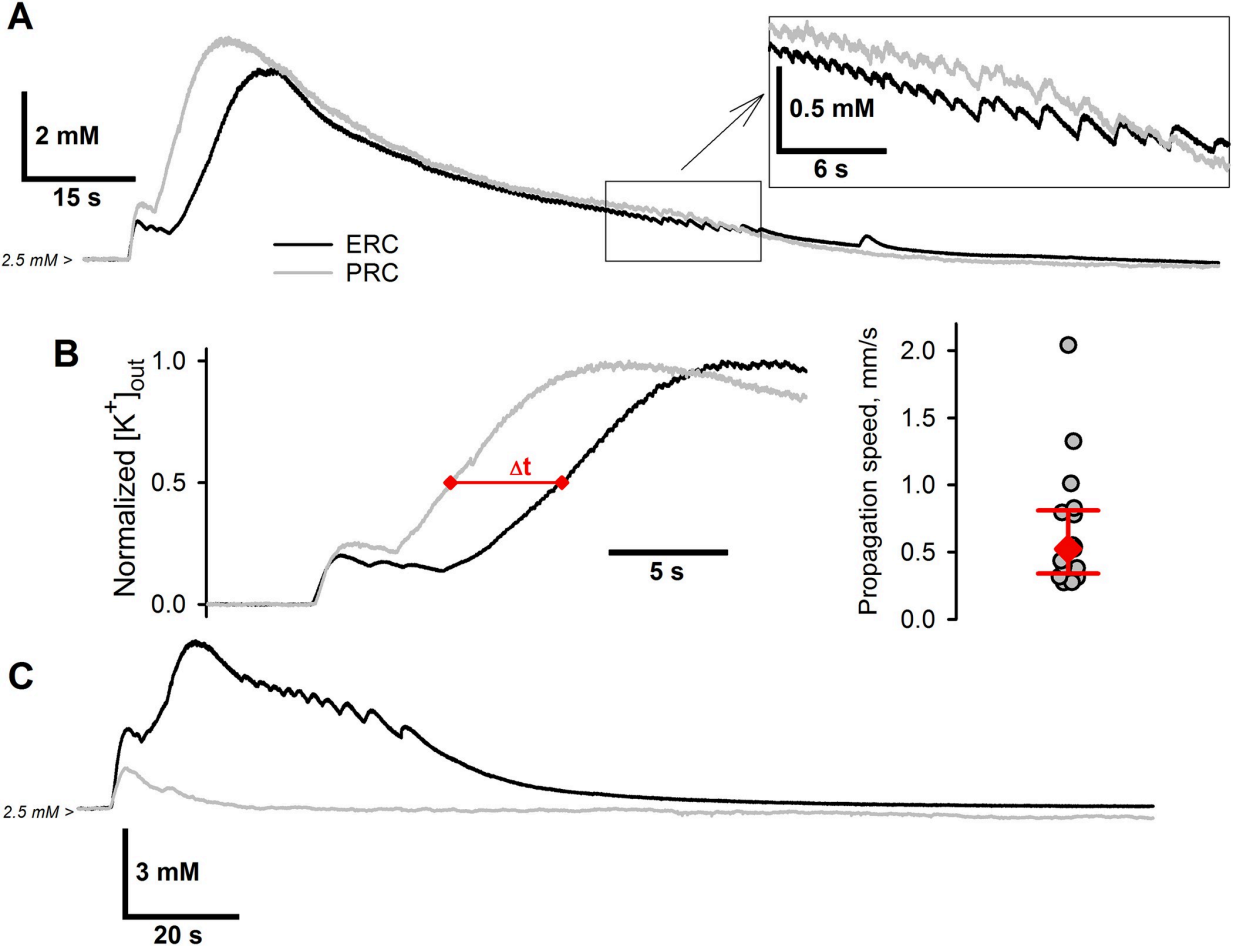
Experimental estimations of the ID propagation speed. (A) The representative paired recordings of [K^+^]_o_ during the IDs at a 2-mm distance between the electrodes. Note the substantial delay of the [K^+^]_o_ rise in PRC relative to ERC. A fragment corresponding to the late-stage discharges is extended in the box. Late-stage discharges produce the almost synchronous K^+^ transients. (B) Left panel: measurement of the delay between normalized K^+^ transients. The time lag between the [K^+^]_o_ rises was measured at a half peak value of [K^+^]_o_. Right panel: the estimation of the ID propagation speed. The red diamond indicates the median value; error bars indicate 75th and 25th percentiles. (C) A representative example of the absence of ID-related K^+^ transient propagation from one cortical area to another in the slice.

As the shape of the K^+^ transients during each ID is relatively standard with its rising phase being related to the recruitment of glutamatergic neurons into the activity, we measured the time delay between rises of K^+^ transients in the ERC and PRC to estimate the propagation speed (Fig 3). The half-maximal [K^+^]_o_ was used as a reference point to measure the delay between the events (Fig 3B), thus ignoring the initial weak component, presumably determined by the potassium ion outflux from synapses activated by long-range connections. The delays between K^+^ transients were comparable to those observed using the patch-clamp method, and the median speed was estimated to be 0.52 mm/s (IQR: 0.34–0.81, *n* = 17 pairs of IDs from eight slices). No significant difference between propagation speed from ERC to PRC and from PRC to ERC was detected (*p* = 0.42, Mann–Whitney *U* test). The recordings, where the ID-related K^+^ transient failed to propagate from one cortical area to another, were not used for speed estimations (Fig 3C). Concluding the presentation of experimental results, we point out that our experiments and similar previous studies mentioned in the Introduction reveal a challenging task to explain the mechanism of slow propagation of ID wavefront with the speed of the order of magnitude of tenths of a millimeter per second.

### Simulation of the ictal front wave

Aiming to reproduce and explain the experimentally observed epileptiform activity, we have constructed a mathematical model (see the Methods section) that describes the main physiological mechanisms underlying interactions between neuronal populations in the cortical tissue. Below, we begin with the description of the simulated phenomenon, then pass to the mechanisms of ID generation and end up with the analysis of ID propagation.

### Phenomenon

**Fig 4:**
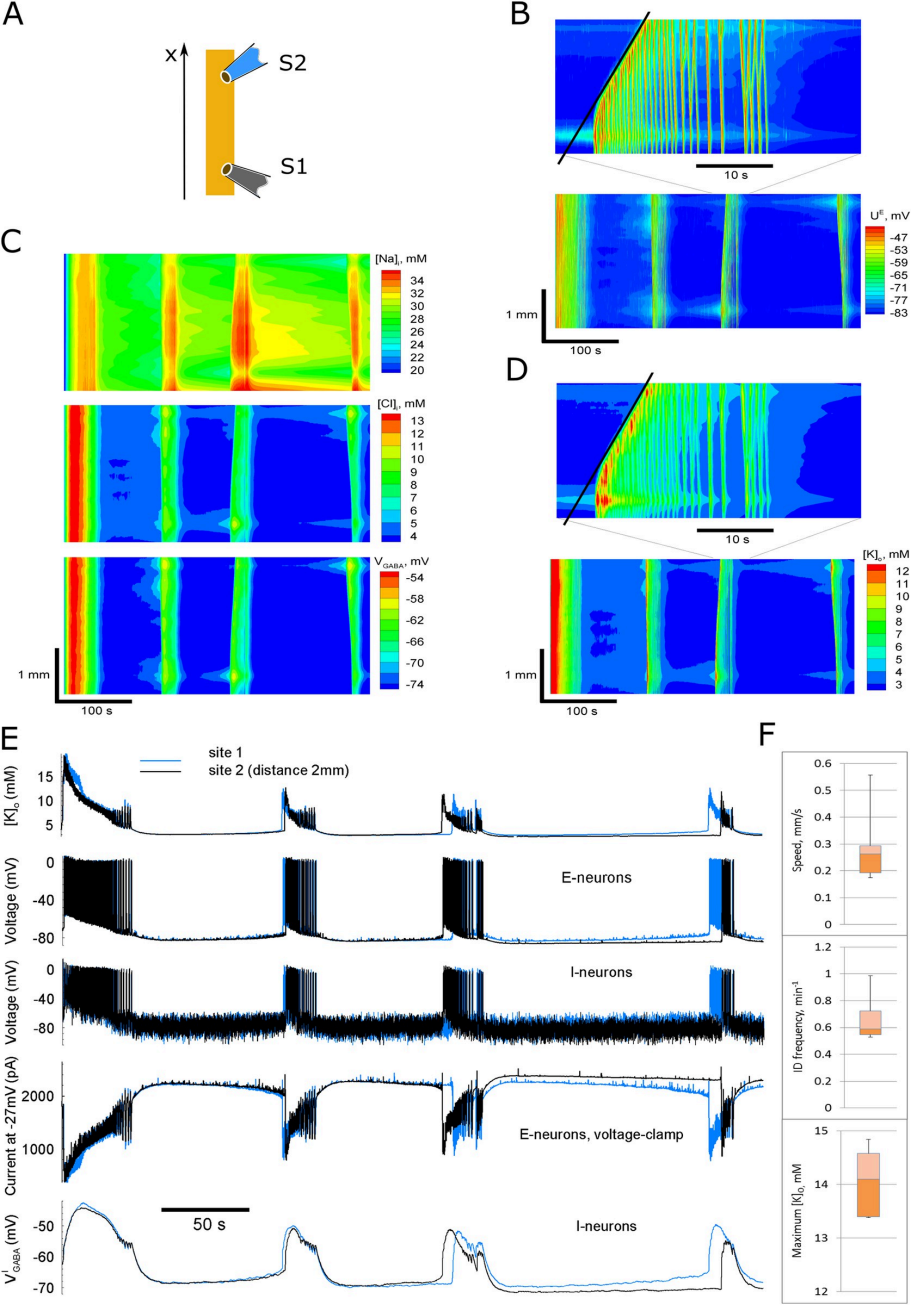
Ictal discharges simulated in the 1-D model. (A) Schematic diagram of the computational domain and the locations of “electrodes” S1 and S2. (B–D) Space-time plots for: (B) the mean membrane potential of E-neurons, (C) the intracellular sodium and chloride ion concentrations, and the GABA reversal potential, (D) the extracellular potassium ion concentration. (E) signals from two sites, S2 (black) and S1 (blue): the extracellular potassium ion concentration, the membrane potential of two remote representative neurons, the current calculated at the “holding” voltage -27mV, and the reversal potential of GABA-A receptors on interneurons. (F) Statistics of the estimated characteristics of the activity, obtained from simulations with the different realizations of noise (3 discharges in 5 simulations, i.e., n = 15): the speed of the wavefronts, the interdischarge frequency, and the maximum value of the extracellular potassium ion concentration reached during IDs. The slope of the black lines in B and D determines the speed of the ictal wavefront. The arrows in bottom panel B point to the ID origination sites.

**Fig 5:**
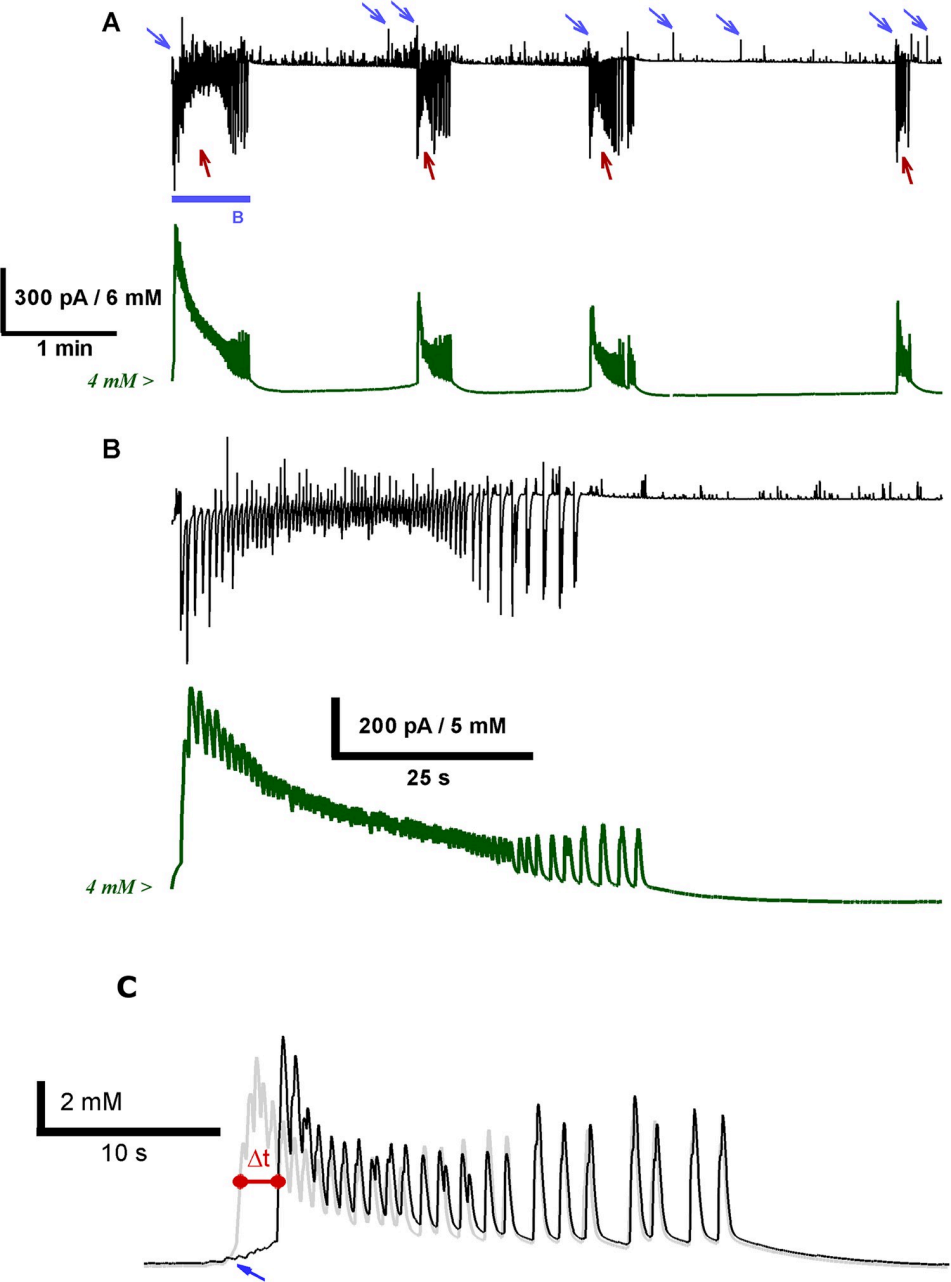
Neuronal and ionic dynamics during ID generation in a simulation are similar to experimental recordings from Figs 1 and 3. (A and B) The signals are the current calculated in the voltage-clamp mode with blocked potassium ion currents (top traces in A and B) and [K^+^]_o_ (bottom). Blue arrows mark the interictal or preictal GABAergic discharges. Red arrows mark the glutamatergic components of IDs. (C) the traces of [K]_o_ at two sites S1 and S2, located at one-fourth of the length from both ends of the simulated “slice” (Fig 4A), in the model that takes into account long-range (all-to-all) connections. The blue arrow marks the initiation of potassium response simultaneously at the two sites. The red notch shows the delay (about 2ms) used to estimate the propagation speed (0.6mm/s for the distance between the electrodes 1.2mm).

We simulated neuronal activity in a spatially extended cortical domain (Fig 4A, yellow domain), thus obtaining the spatial-temporal distributions of the main variable, the membrane voltage, ionic concentrations, etc. (Fig 4B–4D) and the signals from one (Fig 5) or two spatially remote sites S1 and S2 (Figs 4E and 5C). The model reproduces the spontaneous generation of repeating IDs and their spread. Similar to the experimental data, the model generates IDs every few minutes (mean ID frequency was 0.59 s^-1^; Fig 4F) that last for tens of seconds (Figs 4B–4E and 5). At the beginning of each ID, the outward GABAergic current predominates, and the simulated current at the holding voltage of –27 mV exposes short positive, i.e., GABAergic, bursts (Fig 4E, next to bottom; blue arrows in Fig 5A; compare to the experiment in Fig 1A). Then, the inward glutamatergic current prevails, exposing long-lasting negative, i.e., glutamatergic, components (Fig 4E, next to bottom; brown arrows in Fig 5A), similar to what is observed in the experiment (Figs 1 and 2A). These GABAergic and glutamatergic components constitute an ID. Each ID is characterized by the rapid increase in the concentration of the extracellular potassium ions, which peaked in simulations up to 14 mM (Fig 4F). The traces of [K^+^]_o_ at the sites S1 and S2, shown in Fig 4E, have similar shapes. The rapid growth of [K^+^]_o_ is delayed at one of the electrodes. It is in contrast to such traces obtained in the model that takes into account long-range (all-to-all) connections, which reveal a rather simultaneous initiation with weak components (Fig 5C), similar to that in experiments (Fig 3). As discussed below in the paragraph of the Results section “ID propagation in the networks with additional all-to-all connections,” the long-range connections do not affect the bulk of IDs, and thus they are omitted in most of the simulations.

### Mechanism of ID generation

**Fig 6:**
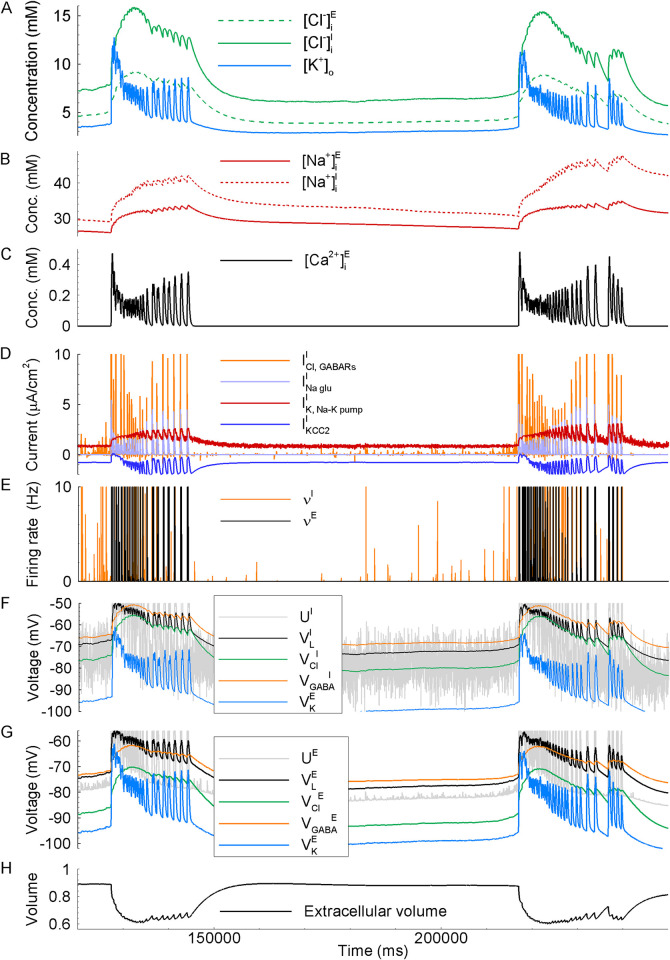
Interneuronal activity triggers IDs in simulation, site S1. For the second and third IDs (as in Fig 4), the following signals are shown (top to bottom): (A) the extracellular potassium and intracellular chloride ion concentrations; (B) the intracellular sodium ion concentrations; (C) the intracellular calcium concentration in E-cells; (D) the GABA-mediated chloride ion current, the glutamatergic sodium ion current, the potassium ion current via the Na^+^/K^+^ pump, and the potassium ion current via the KCC2 cotransporters for *I*-cells; (E) the firing rates of the *E*- and *I*-cells; (F and G) the membrane potential and the reversal potentials of the total leak, chloride ion and GABAergic currents, and the reversal potential of the potassium ion current, for the *E*- and *I*-cells, respectively; (H) the ECS volume.

**Fig 7:**
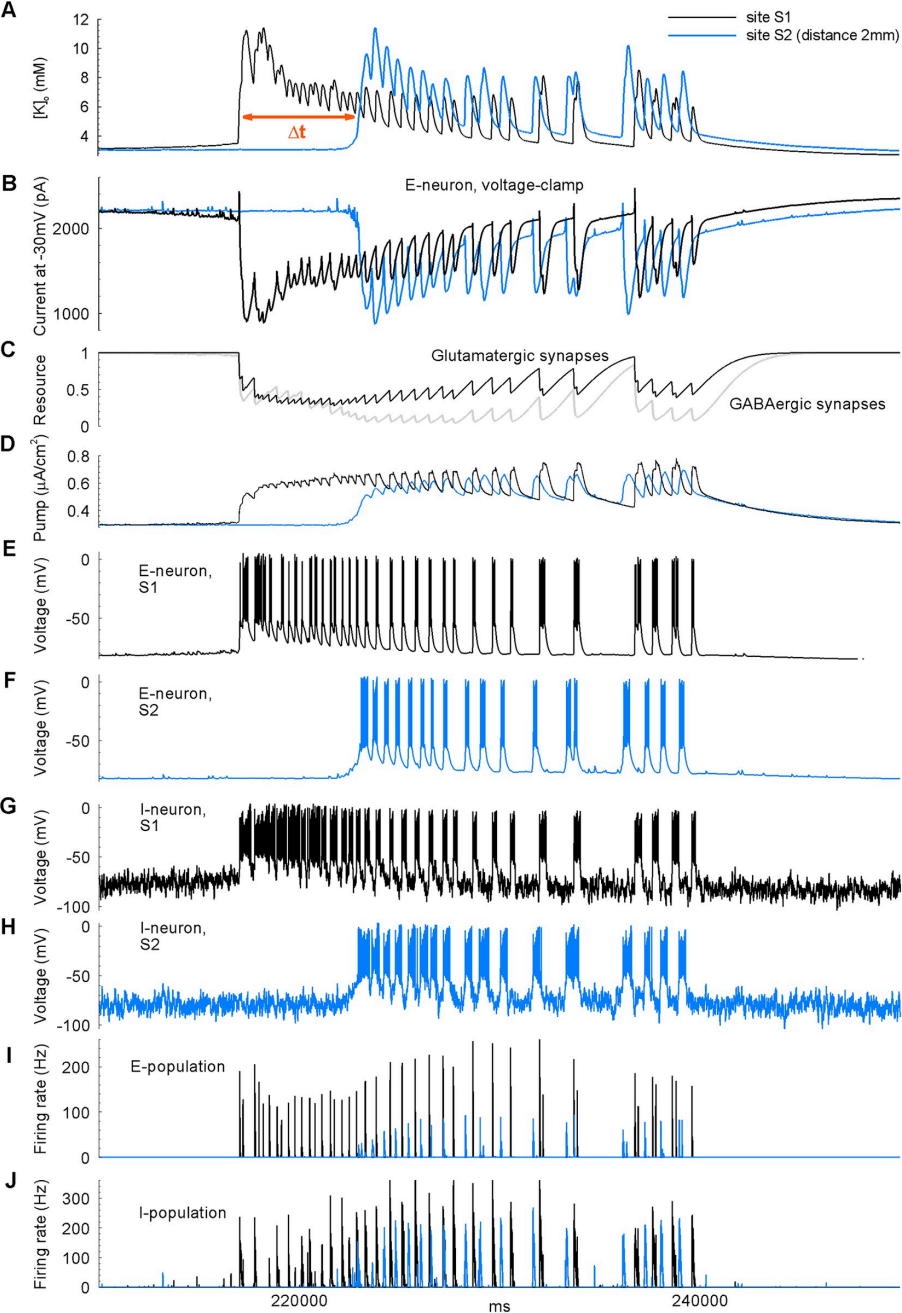
Single ID simulated in the 1-D model. Black–site S1, blue–S2. Red notches mark the interval between IDs registered at the sites S1 and S2.

We observe that in our conditions, interneurons do not inhibit but instead stimulate the pyramidal cells, whose activity then constitutes each new ID. Between IDs, the interictal and preictal GABAergic components seen as positive current events in Fig 5A are determined by spiking of the *I*-neurons, characterized by their firing rate shown in Fig 6E (see also the positive currents in Fig 7B and the *I*-neurons’ firing rate in Fig 7J). The interneurons start to fire spontaneously before the ID (see small initial events in the firing rate trace for the *I*-population in Figs 6E and 7J). The *I*-neurons’ initial firing is determined by the noise that mimics spontaneous synaptic activity, observed in the experiment and caused by increased synaptic transmitter release after 4-aminopyridine application. The enhanced *I*-neurons’ firing increases the frequency of IPSCs (positive current events between IDs in Fig 5A and orange curve for the GABAergic current in Fig 6D).

The chloride movement into and out of cells is described as a sum of the passive conductive flux [31] and fluxes mediated by three proteins: the two cation-chloride-transporters, KCC2 and NKCC1, and the GABA_A_ receptors (see $I_{GABA}^{I}$ in Fig 6D). Their effect leads to a gradual accumulation of chloride ions inside neurons. The GABA reversal potential (Fig 4E, bottom, and Fig 6F) and the leak reversal potential (Fig 6F) increase due to chloride ion accumulation inside the cells. At every GABA-mediated event, the membrane potential tends to approach the GABA reversal potential, which is always above the chloride ion reversal potential (Fig 6F) because of the bicarbonate ion permeability of GABA_A_ receptors. In the case of membrane potential being above the chloride ion reversal potential, the activation of GABA_A_ receptors increases the chloride ion entry into the neurons, even in the absence of depolarization by glutamatergic currents.

The KCC2 transporters diminish the difference between the reversal potentials for chloride and potassium ions. Thus, the elevation of [Cl^–^]_i_ results in the elevation of [K^+^]_o_ and depolarization. The gradual depolarization of neuronal membranes and the depolarizing effect of spontaneous GABA- and glutamate-mediated events lead to excitation and synchronization of some *I*-neurons. The frequency of short bursts of *I*-cell excitation increases (see more significant events in the firing rate trace for the *I*-population in Fig 6E after *t* = 180 s).

The GABAergic activity leads to chloride ion accumulation in *E*-neurons (see the intracellular chloride ion concentration in Fig 6G), raising the reversal potential of GABA_A_ receptor-mediated current and membrane potential. Therefore, *I*-neurons excite *E*-neurons, triggering glutamatergic events (see the *E*-population firing rate in Fig 6E and Fig 7I).

Glutamatergic excitation and elevation of [K^+^]_o_ act as positive feedback, thus maintaining the neural network’s overexcitation. In opposition to glutamatergic drive, the synaptic resource, i.e., the pool of synaptic vesicles ready to release, is depleted (Fig 7C) and interrupts firing, thus splitting ID into a series of bursts, LSDs (Fig 7E).

Sodium and potassium ion currents through voltage-gated and glutamatergic channels elevate [K^+^]_o_ and [Na^+^]^E^_i_ (Fig 6A and 6B). High [Na^+^]_i_ activates the Na^+^/K^+^ pump. The pump provides a hyperpolarizing current and recovers the potassium transmembrane gradient, thus diminishing the positive feedback. It results in the termination of ID. The [Na^+^]_i_ returns to its normal level with some delay, and the pump over-decreases [K^+^]_o_, which results in a pause in neuronal firing and determines the interval between IDs.

**Fig 8:**
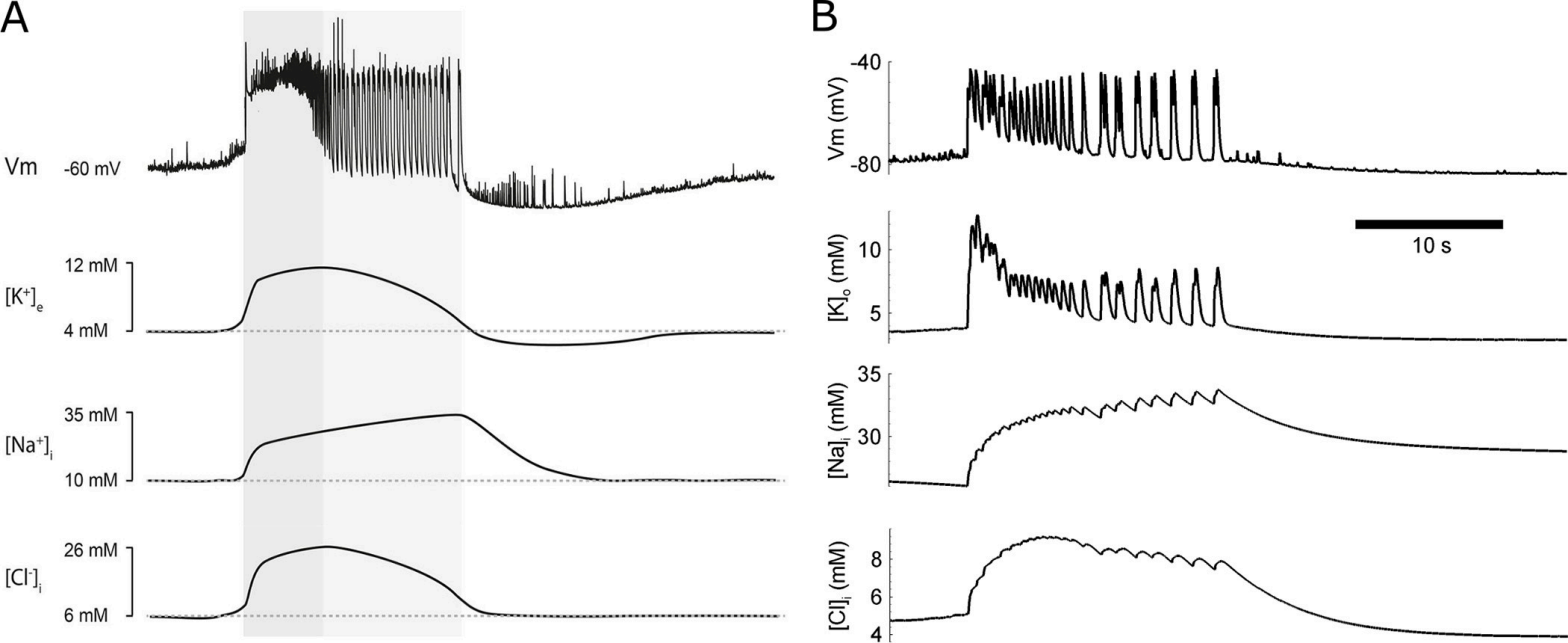
Comparison of simulation with experiment. Neuronal membrane potential and ionic concentration dynamics during a single ID. (A) Experimental “best approximations” based on the available literature, adopted from [32]. The dark and light gray areas mark the tonic and phasic phases of ID. (B) Simulation as in Figs 5–7.

This mechanism of ID generation is consistent with previous studies [21,32,33]. In particular, the dynamics of the neuronal membrane potential and the ionic concentrations is similar to the “best approximations based on the available literature,” presented in [32], as seen from a comparison of the panels of Fig 8. [K^+^]_o_ peaks at the initial phase of ID with an undershoot accompanying the interval between IDs, as in Fig 1A. [Na^+^]^E^_i_ is likely to peak at the end of the ID [18]. [Cl^-^]^E^_i_ reflects the recordings from [33,34]. The aforementioned activation of the Na^+^/K^+^ pump explains the earlier peak of [K^+^]_o_, the later peak of [Na^+^]^E^_i_ and the undershoot of [K^+^]_o_.

### ID propagation

In simulations, as in our experiments, different IDs emerge in distinct parts of the slice. For the simulation shown in Fig 4, the sites of ID origination are marked by arrows in bottom panel B. For instance, the last two IDs (Fig 4B) appear on opposite sides of the “slice.” Except for the duration, the signals recorded in spatially remote sites are similar (Figs 4E and 7). The ID at the leading site lasts longer, such that the ID ceases almost simultaneously at different places, as often observed in experiments [2,35]. At the onset of ID, [K^+^]_o_ and the membrane potential in all types of neurons rise more gradually in the site-follower (Fig 7, blue curves) than in the leading site (black curves). In the later phase of ID, the LSDs appeared almost simultaneously in both sites. Any PIDs that were sometimes observed in the experiments were not present in the simulations.

**Fig 9:**
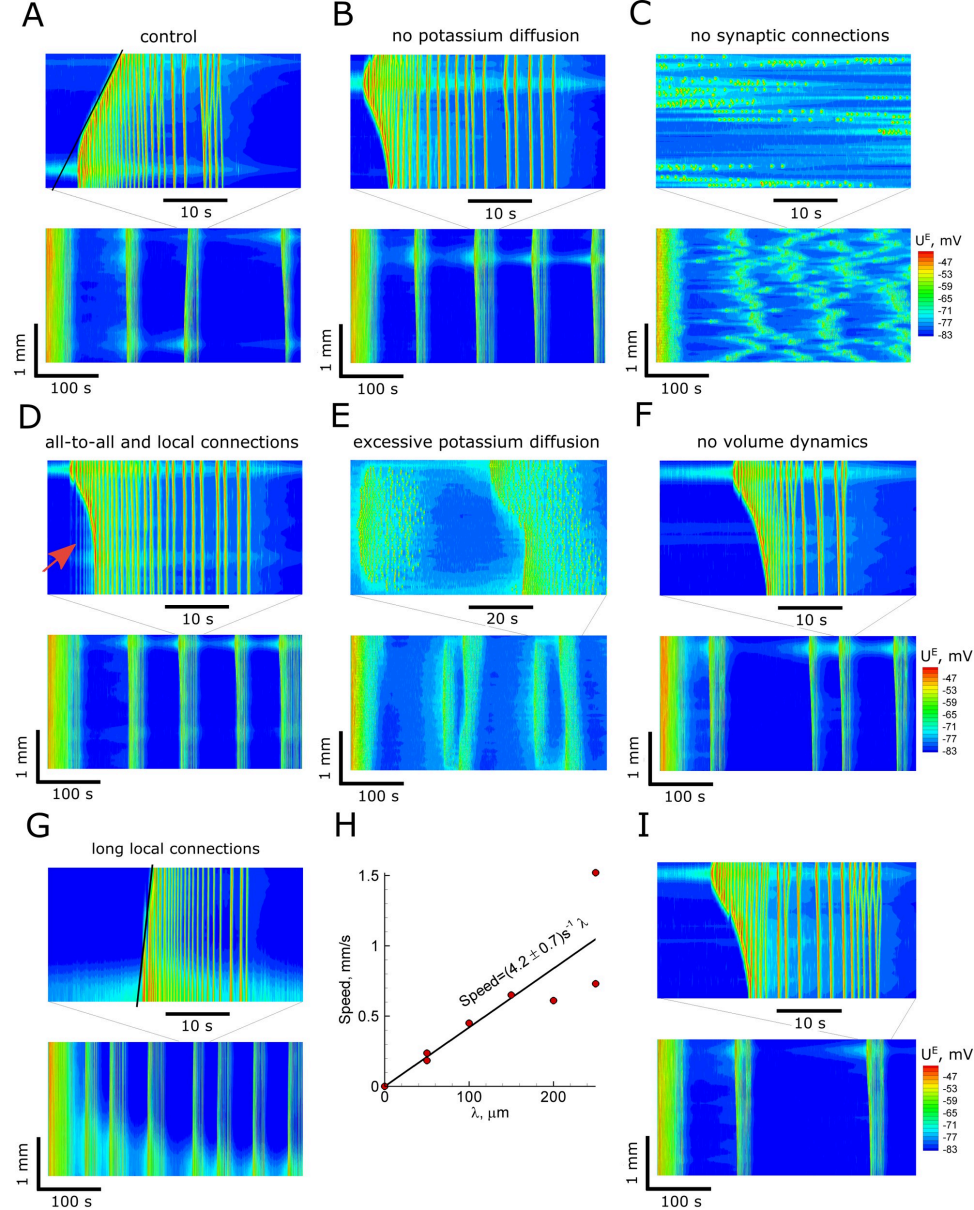
Ictal discharges simulated in the 1-D model with different spatial propagation mechanisms. (A) The control case is as in Fig 4D. (B) Blockade of extracellular potassium ion diffusion does not change the generation of the IDs and their speed. (C) Eliminated spatial spread of synaptic connections results in uncorrelated spatial activity. (D) Preictal bursts emerge in the presence of spatially homogeneous, all-to-all connections. (E) Non-spreading synaptic connections and an artificially increased potassium diffusion coefficient (*D*_1*d*_ = 390 instead of 0.39*μm*^2^/*ms*) result in propagating IDs. (F) Blockade of the volume dynamics increases the irregularity of the ID intervals. (G) The speed of the ID increases with the increased length of local connections (*λ* = 250*μm* instead of 50*μm*). (H) The ID speed is proportional to the length of the connections. (I) The glial buffer increases the intervals between IDs.

While some of the IDs appear almost simultaneously in different locations, i.e., their apparent speed is large, the others propagate with limited speed. Our study focuses on the propagation of IDs at a minimum velocity. A few-second delay is observed between some of the IDs recorded at a 2-mm distance (Fig 4E). For instance, the delay is about 5 s for the third ID shown in Fig 7A. Respectively, the ID wavefront speed is estimated to be 0.4 mm/s, which is a typical minimal speed among different IDs in different realizations of simulations with the fixed control settings (mean value 0.26 mm/s; Figs 7F and 9H). This value within an order of magnitude is consistent with our experimental estimates and the experimental data from other laboratories [3,10,11].

### ID propagation in conditions of no potassium ion diffusion

Our mathematical model includes two mechanisms that may contribute to ID propagation: (i) the diffusion of potassium ions through extracellular space, described by Eq 27 and (ii) the propagation of action potentials through axons, followed by the transmission through synapses and propagation of postsynaptic signals by dendrites, described by the connection profile, Eq 33, with the synaptic conductance equations, Eqs 14–18. To separate the effects of the two mechanisms, we blocked potassium ion diffusion (last term in Eq 27). Still, we observed repeating ictal discharges (Fig 9B) with a pattern that differed slightly but was qualitatively similar to the control case (compare Fig 9B to 9A). The time difference in the ID onsets was similar to that in the control case, suggesting that the ID propagation speed was about 1 mm/s or less. This evidence indicates that the diffusion of potassium ions is insignificant. It indicates that the primary mechanism of ID propagation is based mainly on spatially distributed synaptic connections.

### ID propagation in the networks with non-spreading connections

Next, we tested whether artificial elimination of synaptic connections’ spatial spread has a decisive effect on discharge propagation. For this purpose, we changed the characteristic length of the Gaussian profile of the strengths of the connections *λ* to 0 in Eq 33, thus obtaining *φ*_*i*,*j*_(*t*,*x*) = *ν*^*i*^(*t*,*x*), i.e., the presynaptic firing rate *φ*_*i*,*j*_(*t*,*x*) at any position *x* for the population *j* is now determined by the firing activity *ν*^*i*^(*t*,*x*) of the corresponding neuronal population *i* in the same position *x*, no longer integrating the firing rates from nearby or remote neurons. Simulation has shown a dramatic change in the activity pattern (Fig 9C). Still, at every position *x* at the same time interval 400 s, we observe four or five IDs; however, the discharges are not so synchronized as in the previous simulations. However, there are aligned patterns with a slope corresponding to the extremely slow propagation (between 0.1–0.01 mm/s). These effects seem similar to the spreading depression, which may spread through K diffusion, with almost no synaptic effects.

The dramatic difference between activity patterns in Figs 8A and 9 confirms the primary role of synaptic connections and their spread in wavefront propagation.

*ID propagation in conditions of excessive potassium ion diffusion*. Since the potassium ion diffusion may hypothetically provide the propagation of discharges, in the conditions of eliminated spatial spread of synaptic connections, we increased the diffusion coefficient 1000 times (390 instead of 0.39 μm^2^/ms) but maintaining *λ*→0. The simulation gave the spatially structured pattern shown in Fig 9E. It is consistent with the modeling results by Martinet et al. (2017) [22], where the diffusion coefficient was set to be 10^5^ μm^2^/ms. Nevertheless, such a significant discrepancy in the numbers argues against the potassium ion hypothesis.

*ID propagation in the networks with long connections*. If the potassium ion diffusion’s role is negligible, then the speed of the ID wavefront must depend on the spatial parameter *λ* in the governing equations. We increased *λ* fivefold and obtained the pattern with fast-propagating IDs (Fig 9G). The speed was 1.1 mm/s instead of 0.28 mm/s in the control case (Fig 9A) or 0.35 mm/s in a lack of potassium ion diffusion and short connections (Fig 9B). Hence, the connection length is the major factor that determines the speed of IDs. Neglecting potassium ion diffusion, the length of connections is the only spatial scale in the model, which enters through Eq 33. Hence the ID speed is just proportional to *λ*. Estimating the speed of the slowest waves in simulations with different values of *λ* and different realizations of noise (Fig 9H), we obtained the coefficient of the proportionality to be 4.2 ± 0.7 s^-1^.

### ID propagation in the networks with additional all-to-all connections

In the previous simulations, we considered Gaussian-like profiles of spatially-extended connections without long-range connections, which may affect ID propagation. We also suggest that the effect of long-range connections explains the appearance of PIDs observed in the experiments but missed in the simulations mentioned above. To test these hypotheses, we added homogeneous global connections to the Gaussian profile. To this end, a fraction of the presynaptic rate (0.2) was set to be proportional to the firing rate averaged across the entire domain. As a result, the activity pattern remained approximately the same as in the control case (Fig 9D), but weak pre-ictal bursts became evident (see the arrow in Fig 9D). The PIDs are accompanied by the weak raise of [K]_o_, initiated almost immediately in the whole “slice,” as seen from the traces at two sites (Fig 5C). The weak [K]_o_ growth is determined by the potassium outflux from the synapses activated by the long-range connections. Since the fraction of these connections is small, the weak raise of [K]_o_ does not affect the bulk of ID. Thus, the long-range connections do not crucially affect the ID propagation but determine PIDs.

### The effect of volume dynamics

The volume of extracellular space (ECS) changes significantly during IDs [36], which affects the discharges [19] and hypothetically might affect the ID propagation. Volume dynamics provide feedback for the modulation of ionic concentrations. A change in ionic balance results in a change in osmolarity and, consequently, volume change, which in turn primarily affects the extracellular concentrations. We describe the volume dynamics with Eq 32, and its effect is considered for the extracellular ion concentrations in Eqs 27–29. The ECS volume is much smaller than the intracellular volume, which is characterized by the ratio *β*. This ratio is close to the reverse ECS volume fraction estimated from the experiments. The ECS volume fraction ranges from 5% to 36% [37,38]. An effective ECS volume fraction is even less if we exclude the contribution of large reservoirs of ECS into the estimated value, so we set *β =* 10 in contrast to 7 from the previous modeling study [39]. Since the ECS volume is much smaller than the intracellular volume, any water flux out or inside cells leads to a more significant change of the ECS volume than of the intracellular volume. Therefore, it has a stronger effect on extracellular than intracellular ionic concentrations. The ECS volume reduces after a single ID at almost 50% and gradually recovers between IDs (Fig 6H), which is consistent with the experimental observations [36]. Such a volume change affects the extracellular ionic concentrations even if the intracellular concentrations remain constant. To check the ECS volume change effect on ID generation, we performed a fixed volume simulation (Fig 9F). The simulation shows no significant changes in the frequency and speed of the IDs. The magnitude of modulation of [K^+^]_o_ and other variables during the IDs are almost the same in the simulations with dynamic and fixed ECS volume, except for the [Cl^-^]_o_ changes, which are bigger in the case of fixed volume. In this case, ID generation is more irregular, observed as a larger difference in interdischarge intervals (compare Fig 9F to 9A).

### The effect of glia

To consider the effect of glia, we took into account a glial buffer in the form from [40]. Simulations revealed a quite expected increase of the intervals between IDs but no evident effects on the ID propagation (compare Fig 9I to 9A).

## Discussion

### The phenomenon of epileptiform discharge propagation

We studied seizure propagation properties using an *in vitro* model of epileptiform activity and mathematical modeling. With electrode recordings made at two distant sites of the cortex, we found an ID propagation speed of less than 0.5 mm/s. In simulations, we reproduced the regime of repeating ID generation and propagation with speed close to the experimental estimates within an order of magnitude. Our experimental observations and simulations are consistent with data in patients and results obtained from *in vivo* and *in vitro* animal models. For instance, Martinet et al. (2015) [41] used invasive subdural electrode arrays covering a broad area of the cortex (8 × 8 cm) of patients with pharmacoresistant epilepsy to visualize seizure spread and observe waves of cortical region recruitment. They measured a mean recruitment speed of about 4 mm/s. Our estimations (Figs 3B and 4F) are also consistent with the previous EEG and fMRI studies in human patients, where propagation speeds varied from 0.2 to 10 mm/s [42–45]. The multiunit ECoG recordings showed the propagation to be 0.83 mm/s [7]. In *in vitro* models of epileptic activity with the zero magnesium (0 Mg^2+^) solutions, the speed varied from 0.1 to 1.34 mm/s [12,46,47].

The speed of the IDs was crucially slower than that of the SDs, including LSDs (referred to as the afterdischarges in other studies [35]) and interictal discharges, which were measured to be > 30 mm/s [2,3,12]. This difference is also evident from our experiments and simulations and corresponds to our previous study on SDs [6]. The ID propagation is much lower than the axonal conduction velocities found in the brain, e.g., for mossy fibers (300 mm/s) [48]. From the other extreme, the ID speed is much faster than that of cortical spreading depression observed in migraine (3 mm/min) [49]. Our simulation with non-spreading connections (Fig 9C) seems to be close to the case of cortical spreading depression. It shows similar speeds of discharges propagating purely by means of potassium diffusion. However, this issue requires further investigation.

Typically, in the late stage of an ID, the LSDs recorded at spatially remote sites are highly correlated [35]. They look synchronized on the time-scale of an entire ID because of their high speed. So, naturally, the IDs recorded at different sites typically cease simultaneously. That is why the ID spread studies reveal the tendency of IDs to be shorter at the locations where the ID front appears later [41], as also seen in our examples.

In our experiments, IDs were preceded by PIDs, which were not seen in the control simulation. However, after consideration of the long-range connections, such PIDs have been revealed in simulations, which clarifies the origin of the discharges: the PIDs were reflected through the long connections from the leading zone of ID generation.

### Mechanism of ID generation

The ID origination scenario in our model supports the GABAergic hypothesis of seizure initiation [50,51]. This hypothesis proposes that synchronous activation of inhibitory interneurons underlies the inciting events that result in a seizure. In our case, the involvement of pyramidal cells goes through chloride accumulation [33,52] rather than through post-inhibitory rebound excitation [51,53]. The synchronous activation of interneurons is also supported by the chloride ion accumulation in these cells. That is why the ionic dynamics play a crucial role in the initiation of ID generation and maintenance of this activity. Therefore, it was essential to describe it in detail in our model. Briefly, intracellular chloride and extracellular potassium ion concentrations elevation provides positive feedback during a single ID generation. In contrast, the intracellular sodium ion accumulation does negative feedback terminating each ID through the activation of the sodium-potassium pump [54]. Considering the fast negative feedback through the short-term synaptic depression and the calcium-dependent potassium channels, we obtain IDs in the form of clustered bursts of spikes. At the peak of depolarization during ID, neurons may show tonic firing or fall into the depolarization block. In total, the scenario is consistent with classical observations [1].

### Mechanisms of ictal wavefront propagation

**Fig 10:**
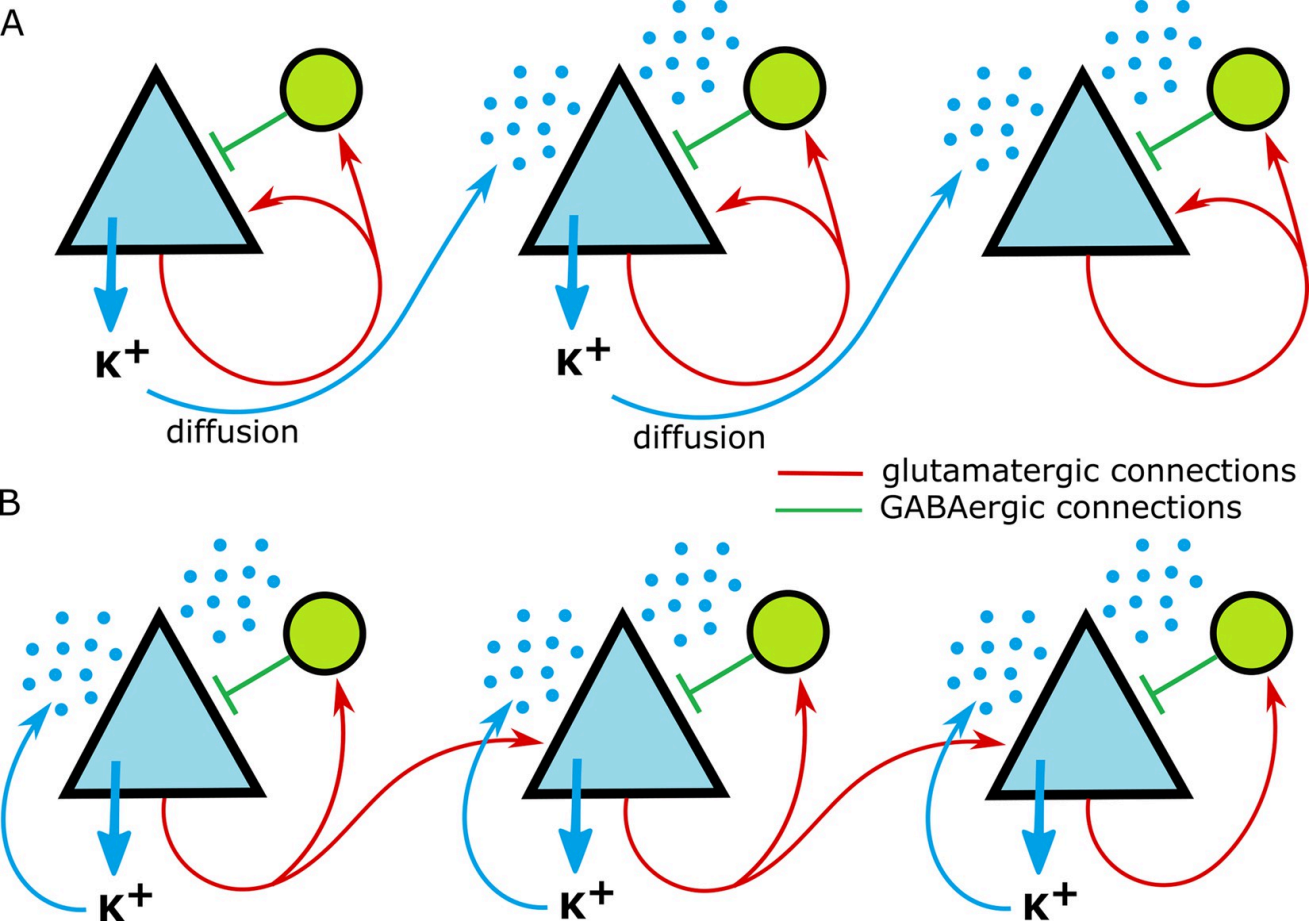
Two alternative mechanisms of ID propagation. (A) The local extrusion and the diffusion in the extracellular space of potassium ions result in potassium ion-mediated depolarization of distant pyramidal cells (blue triangles) and interneurons (green circles). (B) Local extrusion of potassium ions supports excitation of nearby pyramidal cells and interneurons. The pyramidal cells fire their spikes, which propagate through axons and activate glutamatergic receptors, thus providing excitation and extrusion of the potassium ions through voltage-gated and glutamatergic channels, which trigger ID in new locations.

In the literature, several mechanisms of ID and SD propagation were suggested, the first two of which we considered in the present paper (Fig 10): (i) synaptic transmission between neurons, including spike propagation through axons and passive conduction of postsynaptic signals through dendritic neuronal branches [23,24]; (ii) the diffusion of potassium ions, as a substantial transient increase in extracellular potassium ion concentration is observed during IDs [1,22,25,26]; (iii) the ephaptic interactions of neurons through an electric field [55]; and (iv) the electrodiffusion of potassium or glutamate ions in the extracellular space [56].

As shown with modeling [6], IID and LSD propagation is determined by neuronal branching and synaptic transmission (the first mechanism) only. However, regarding IDs, with only this fast propagation mechanism being considered, it was unclear how the ictal wavefront travels within cortical tissue at such a slow speed as less than 1 mm/s. Moreover, the wavefront of potassium ion elevation usually accompanies an ID [1], as observed in our experiments. This observation points to the critical role of potassium ion elevation and its depolarizing effect. However, whether the potassium ions spread by diffusion is the cause of the ictal front’s propagation (the second mechanism) was an open question. The speed of the potassium ion waves due to ionic diffusion is a constraint made by the effective diffusion coefficient that is affected by the tortuosity and estimated to be less than 1 mm^2^/ms [57–59]. It is uncertain if the potassium ion diffusion-based hypothesis and simulations [22] are consistent with the experiments. An artificially increased diffusion coefficient may provide speeds comparable to those of IDs, as in [22], where the coefficient was five orders of magnitude higher than the actual values (1 cm^2^/s). Recently, direct evidence concerning the lack of potassium ion diffusion effect on ID propagation from the hippocampus to the neocortex has been provided [60]. The authors reasoned that if activity in the follower territory is triggered by a rise in the number of potassium ions diffused from the other site, then for those events, the rise would appear to occur significantly earlier relative to the local firing. In fact, they have found no significant difference in the latencies, suggesting that the entrainment of neocortical events by the hippocampal activity in their preparation does not happen via the diffusion of potassium ions.

The third mechanism based on ephaptic interactions is known to determine the propagation of slow periodic epileptiform activity in the conditions of absent synaptic interactions [55]. However, it is generally too weak: the extracellular electrical potential with a typical magnitude (about 1 mV) can hardly increase the transmembrane potential up to its threshold value, which is usually much more than a few millivolts above the resting level. Nevertheless, the ephaptic interactions are supposed to trigger excitation in the case of reduced extracellular space [61]. The fourth mechanism of electrodiffusion is also too weak. As estimated earlier [28], the electric field gradients at an order of 2 mV per 100 μm may provide the speed of potassium ions of a few μm/s, which is negligibly small. Therefore, the last two mechanisms can be excluded from consideration. Thus, the present study was aimed to distinguish between the first two mentioned mechanisms of ID propagation through electrophysiological recordings and modeling.

Our simulations have helped to distinguish between the first two mechanisms (Fig 10). We have found that the potassium diffusion-based mechanism (Fig 10A) leads to a much lower speed of propagation, which refutes this hypothesis. On the contrary, the neuronal branching-based mechanism (Fig 10B) determines the ictal front rate; thus, it plays a more important role. In this mechanism, the elevation of [K^+^]_o_ is also essential for the excitation; however, it might be localized, i.e., it follows the propagation of synaptic activity. These findings are consistent with our previous simplified modeling study [28]. We suggest that this mechanism is also the major one that explains the above-mentioned observations made in the *in vitro* models and patients; though, the large-scale recordings with EEG or fMRI electrodes may also reflect the contribution of the cortico-thalamo-cortical loops [41,62–66].

### Mechanism of propagation of SDs, which are much faster than IDs

As described in our previous study of SDs with modeling and electrophysiological tools [6], the mechanism of SD propagation includes only synaptic connections. It does not necessitate the ionic dynamics, though the pathologically impaired, quasi-constant level of intracellular chloride ion concentration is required for the generation of SDs. In our present simulations, the ionic concentrations were dynamic, but the SD mechanism was the same. Thus, it validates the conclusion of our previous study, stating that the SD propagation is independent of the ionic dynamics. In contrast, the ID propagation necessarily involves at least the dynamic change in extracellular potassium ion concentration. Consequently, the ID front propagates much slower than SDs, at the speed of a few tens of mm/s, so on the time scale of an entire ID, SDs are observed as almost synchronous events [2,3].

### Factors affecting ID propagation

The present detailed model has revealed the main factors affecting propagation and some of the inefficient ones. The most influential parameter was the length of the connections. In our simulations, it increased the ID speed almost proportionally. It may also explain the bulk difference between the studies in humans and rodents: the typical length of collaterals in human brains is much longer. On the contrary, modulation of the potassium ion diffusion coefficient within its physiological range was inefficient. The volume dynamics and the glial buffering also did not contribute significantly to the ID propagation.

In conclusion, our study distinguishes between two primary hypotheses on the mechanisms of ictal wavefront propagation. It highlights the role of conventional signal propagation through neuronal branches. This axo-dendritic propagation is accompanied by positive feedback excitation provided by the elevation in the level of extracellular potassium ions that are released from excited neurons through voltage-gated and active glutamatergic receptors. These results are of certain importance to novel treatments against epilepsy.

## Methods

### Ethics statement

Three-week-old male Wistar rats (n = 15) were used in this study. The animals were kept under standard conditions with free access to food and water. All animal procedures followed the guidelines of the European Community Council Directive 86/609/EEC and were approved by the Sechenov Institute of Evolutionary Physiology and Biochemistry Bioethics Committee.

### Brain slice preparation

The rats were sacrificed via decapitation, and their brains were removed rapidly. The brain slice preparation method has been described previously (Amakhin et al., 2016; Chizhov et al., 2019). A vibrating microtome (Microm HM 650 V; Microm, Germany) was used to cut horizontal 350-μm-thick slices that contained the hippocampus and the adjacent cortical regions (including the entorhinal cortex (ERC) and the perirhinal cortex (PRC)). Artificial cerebrospinal fluid with the following composition (in mM) was used: 126 NaCl, 24 NaHCO_3_, 2.5 KCl, 2 CaCl_2_, 1.25 NaH_2_PO_4_, 1 MgSO_4_, and 10 dextrose. The artificial cerebrospinal fluid was aerated with a gas mixture of 95% O_2_ and 5% CO_2_. All chemicals used to prepare the solutions were purchased from Sigma-Aldrich (St. Louis, MO, USA) unless stated otherwise. 1–2 slices per rat were used for the experiments.

### *In vitro* model of epileptiform activity

Epileptiform activity in rat brain slices was induced using a pro-epileptic solution (Amakhin et al., 2016; Chizhov et al., 2017, 2019), containing the following (in mM): 126 NaCl, 24 NaHCO_3_, 2.5 KCl, 2 CaCl_2_, 1.25 NaH_2_PO_4_, 0.25 MgSO_4_, 10 dextrose, 0.05 4-aminopyridine. Perfusion of slices with pro-epileptic solution results in an activity similar for ERC and PRC.

### Whole-cell patch-clamp recordings

The recordings were performed at 30°C. Pyramidal neurons in the deep layers of the medial ERC and layer 3 of PRC were visualized using a Zeiss Axioscop 2 microscope (Zeiss, Oberkochen, Germany) equipped with differential interference contrast optics and a video camera (Grasshopper 3 GS3-U3-23S6M-C; FLIR Integrated Imaging Solutions Inc., Wilsonville, OR, USA). Patch electrodes (3–5 MΩ) were pulled from borosilicate glass capillaries (Sutter Instrument, Novato, CA, USA) using a P-1000 pipette puller (Sutter Instrument, Novato, CA, USA). A cesium methanesulfonate-based pipette solution (composition in mM: 127 CsMeSO3, 10 NaCl, 5 EGTA, 10 HEPES, 6 QX314, 4 ATP-Mg, and 0.3 GTP; pH adjusted to 7.25 with CsOH) was used for voltage-clamp recordings. For current-clamp recordings, a potassium gluconate-based pipette solution was used, in mM: 136 K-Gluconate, 10 NaCl, 5 EGTA, 10 HEPES, 4 ATP-Mg, and 0.3 GTP; pH adjusted to 7.25 with KOH. Whole-cell recordings were performed using a Multiclamp 700B (Molecular Devices, Sunnyvale, CA, USA) patch-clamp amplifier and an NI USB-6343 A/D converter (National Instruments, Austin, TX, USA) using WinWCP 5 software (University of Strathclyde, Glasgow, U.K.). The data were filtered at 10 kHz and sampled at 20 kHz. In all cells included in the sample, access resistance was less than 15 MΩ and remained stable (≤20% increase) across the experiment. The liquid junction potential was compensated offline for the voltage-clamp recordings by subtracting 7 mV.

### Extracellular potassium ion concentration recordings

Recordings of the extracellular potassium ion concentration were performed using the monopolar K^+^-selective microelectrodes [60]. The pipettes were pulled from borosilicate glass (Sutter Instrument). The pipettes’ interior surface was exposed to hexamethyldisilazane vapor (Sigma-Aldrich) at 220°C for 90 min. The pipettes were then backfilled with 100 mM KCl solution. A small volume of the K^+^ sensor (Potassium Ionophore I, cocktail A; Sigma-Aldrich, cat. no. 99311) was taken into the salinized pipette’s tip using slight suction. The recording of electrode voltage was performed using the Multiclamp 700B patch-clamp amplifier in current-clamp mode. We checked the stability of the electrodes at the start and end of each recording. Data from unstable electrode recordings were discarded. The extracellular K^+^ concentration ([K^+^]_o_) at a given moment (t) was calculated from the electrode voltage, (V(t)), as follows:

$${\left[K^{+}\right]}_{o}(t)=2.5\;e^{S*V(t)},$$

where *S* is the scaling factor, which was estimated by applying solutions with different [K^+^]_o_ at the tips of ionophore-filled electrodes using a fast application system (HSSE-2/3, ALA Scientific Instruments Inc., USA).

In all electrodes tested, the scaling factor was within a small range (0.043–0.045), so for all obtained recordings, *S* was set equal to the average value of 0.044mV^-1^.

### Experimental data analysis and statistics

The data analysis was performed using custom software written in Wolfram Mathematica 12 (Wolfram Research, Champaign, IL, USA). To estimate SLE propagation speed, a time lag between the corresponding K^+^ transients in ERC and PRC was utilized. The time lag between the [K^+^]_o_ rises was measured at half the peak amplitudes. The characteristic distance was about 2 mm. The upper limit of ID speed propagation was calculated as a ratio of the distance between recorded neurons and the time lag. 2–4 IDs from each slice were recorded for speed estimation.

Sigmaplot 14 (Systat Software Inc., San Jose, CA, USA) was used for the statistical analysis of the results. Dixon’s Q-test (at the 95% confidence level) was used to reject outliners. The Kolmogorov–Smirnov test was employed for the evaluation of the normality of sample data. For data that passed the normality test, the results were expressed as mean ± standard error of the mean. Otherwise, the results are expressed as the median and 25%-75% interquartile range (IQR).

### A CBRD approach for populations of pyramidal neurons and interneurons

The model for connected populations uses a one-population model as a building block. Such a population receives input signals as synaptic conductances, which are determined by the presynaptic firing rates. The population’s output signal is the firing rate, which affects other populations’ presynaptic firing rates, as described by the equation of neuronal activity propagation. The population activity is also affected by the gradients of ionic concentrations, determining the ionic channels’ driving forces.

**Fig 11:**
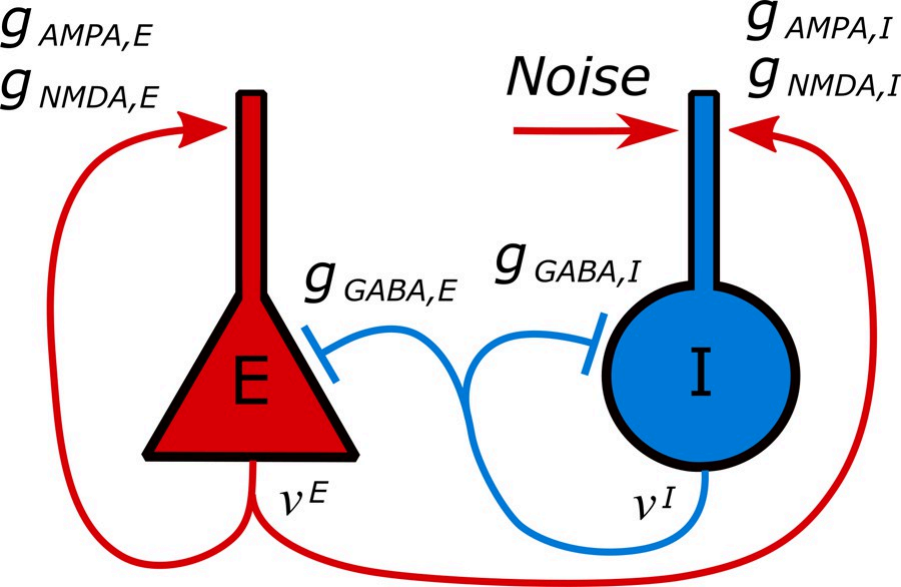
A schematic diagram of the connections between the modeled excitatory and inhibitory populations.

The synaptically interacting neuronal populations model is based on our previous study [21]. We consider excitatory and inhibitory neuronal populations, denoted by indices *E* and *I*, respectfully, which are distributed in the 1-D space of the *x*-coordinate and connected by the synaptic AMPA, GABA, and NMDA receptors (Fig 11).

### CBRD approach for a single population of neurons

The mathematical description of every single population is based on the probability density approach [67], namely, the CBRD approach [68]. This approach considers a population of an infinite number of Hodgkin–Huxley-like neurons receiving both a common input and an individual noise input for each neuron. In any arbitrary case of transient or steady-state stimulation, such a population’s firing rate is approximated with a system of equations in partial derivatives, 1-D transport equations. The equations govern an evolution of neuronal states distributed in the phase space of the time elapsed since the last spike, t*. The equations include the Hodgkin–Huxley equations for the membrane voltage and gating variables, parameterized by t*, and the equation for the neuronal density in t*-space, *ρ*^*p*^(*t*,t*), where the index *p* substitutes for *E* or *I*. The output characteristic of the population’s activity is the firing rate *ν*^*p*^(*t*), which is equal to *ρ*^*p*^ in the state of a spike, t* = 0. According to our previous work, the particular form of the equations written below is for regular-spiking pyramidal cells [69,70].

Basic neurons are single-compartment ones with the membrane voltage *U*^*p*^(*t*,t*). Approximations of voltage-gated ionic currents are based on the neocortical pyramidal cell model from [71], including for *E*-neurons the calcium dynamics and calcium-dependent potassium current that provides an effect of slow spike timing adaptation. Parameterized by t*, the governing equations are as follows:

$$\frac{\partial \rho^{p}}{\partial t}+\frac{\partial \rho^{p}}{\partial t^{*}}=-\rho^{p}H(U^{p},g_{tot}^{p}),$$ (1)

$$\begin{array}{l} C(\frac{\partial U^{p}}{\partial t}+\frac{\partial U^{p}}{\partial t^{*}})=-g_{L}(U^{p}-V_{L})-I_{DR}-I_{K-Ca}+I_{noise}\\ \;+g_{GABA,p}(t)(V_{K}-U^{p})+g_{AMPA,p}(t)(V_{AMPA}-U^{p})+g_{NMDA,p}(t)(V_{NMDA}-U^{p}) \end{array}$$ (2)

where $g_{tot}^{p}(t,t^{*})$ is the total conductance, including the leak, voltage-gated and synaptic conductance $g_{syn}^{p}(t,U_{}^{p})=g_{AMPA,p}^{}(t)+g_{NMDA,p}^{}(t,U_{}^{E})+g_{GABA,p}^{}(t)$.

### Hazard function

The source term in the Eq 1 is the hazard function *H*. This function is defined as the probability to generate a spike for a single neuron that is actually in the state characterized by known neuronal state variables such as the mean membrane potential and gating variables and noise. The approximation of the hazard function *H* has been obtained for the case of white noise [69] and color noise [70] as a function of *U*(*t*) and $g_{tot}^{E}(t,t^{*})$, and parameterized by the noise amplitude in the resting state $\sigma_{V}^{0}$, the spike threshold voltage *V*_*th*_, and the ratio of the membrane time constant *τ*_*m*_ = *C*/*g*_*tot*_ to the noise time constant *τ*_*Noise*_, i.e. *k* = *τ*_*m*_/*τ*_*Noise*_:

H(U)=A+B, (3)

$$A=\frac{1}{\tau_{m}}e^{0.0061-1.12T-0.257T^{2}-0.072T^{3}-0.0117T^{4}}(1-{\left(1+k\right)}^{-0.71+0.0825(T+3)}),$$

$$B=\sqrt{2}{\left[-\frac{dT}{dt}\right]}_{+}\sqrt{\frac{2}{\pi }}\frac{\exp(-T^{2})}{1+\mathrm{erf}(T)},\;T=\frac{V_{th}-U}{\sqrt{2}\;\sigma_{V}}\sqrt{\frac{g_{tot}^{E}}{g_{L}}},$$

where *T* is the membrane potential relative to the threshold, scaled by the noise amplitude *σ*_*V*_, which increases with the synaptic conductance: $\sigma_{V}=\sigma_{V}^{0}\sqrt{1+g_{syn}^{}/g_{L}^{}}$. The term *A* is the hazard for a neuron to cross the threshold because of noise, derived analytically [69] and approximated by exponential and polynomial for convenience; *B* is the hazard for a neuron to fire because of depolarization due to deterministic drive, i.e., the hazard due to drift in the voltage phase space. Note that the *H*-function is independent of the basic neuron model and does not contain any free parameters or functions for fitting to any particular case. Thus, *H*-function is the same for excitatory and inhibitory populations.

### Voltage-dependent channels

The set of ionic currents includes the voltage-dependent potassium currents *I*_*DR*_ and *I*_*A*_ responsible for spike repolarization, the slow potassium current *I*_*M*_ that contributes to spike frequency adaptation and the potassium current *I*_*K*−*Ca*_, dependent on calcium dynamics and contributing to slow spike frequency adaptation. Approximating formulas for the currents *I*_*DR*_ and *I*_*K*−*Ca*_ are taken from [71].

The voltage-dependent potassium current *I*_*DR*_:

$$I_{DR}(U,t,t^{*})={\bar{g}}_{DR}n^{4}(t)(U(t)-V_{K}),$$ (4)

$$\frac{\partial n}{\partial t}+\frac{\partial n}{\partial t^{*}}=\alpha (U)(1-n)-\beta (U)n,$$ (5)

α=−0.032(U+48)/(exp(−(U+48)/5)−1),

β=0.5exp(−(U+53)/40),

The voltage-dependent potassium current *I*_*M*_:

$$I_{M}(U,t,t^{*})={\bar{g}}_{DR}n_{M}(t)(U(t)-V_{K}),$$ (6)

$$\frac{\partial n}{\partial t}+\frac{\partial n}{\partial t^{*}}=\alpha (U)(1-n)-\beta (U)n,$$ (7)

α=−0.0001(U+30)/(exp(−(U+30)/9)−1),

β=0.0001(U+30)/(exp((U+30)/9)−1),

The adaptation current *I*_*K*−*Ca*_:

$$I_{K-Ca}(U^{E},t,t^{*})={\bar{g}}_{AHP}\;n^{2}(t)(U(t)-V_{K}),$$ (8)

$$\frac{\partial n}{\partial t}+\frac{\partial n}{\partial t^{*}}=\alpha (U){\left[Ca^{2+}\right]}_{i}^{2}(1-n)-\beta (U)n,$$ (9)

$$\alpha =2000mM^{-2}ms^{-1},\;\beta =0.002ms^{-1}$$

where [*Ca*]_*i*_ is the intracellular calcium concentration specified below.

### Boundary conditions

According to the conservation of the number of neurons in a population, the firing rate is calculated as a sink of neurons from their state t* due to spiking, *ρ*^*p*^(*t*,t*)*H*(*U*^*p*^(*t*,t*), integrated over the whole phase space, i.e.

$$\nu^{p}(t)\equiv \rho^{p}(t,0)=\int_{+0}^{\infty }\rho^{p}(t,t^{*})H(U^{p}(t,t^{*}))dt^{*}.$$ (10)

It is the boundary condition for Eq 1.

The spike duration is taken into account by introducing the time interval 0<t*<Δ*t*_*AP*_ during which the voltage and the gating variables are fixed to their reset values. It defines the boundary conditions for Eqs 2–7 at t* = Δ*t*_*AP*_ which are as follows:

$$U^{E}(t,\Delta t_{AP})=V_{reset}$$ (11)

$$I_{DR}:\;n(t,\Delta t_{AP})=0.5$$ (12)

The reset values for the fast gating variables in Eqs 10–12 were obtained with the basic single neuron model. With a rather arbitrary input providing a spike, these values were measured at the moment of a voltage maximum at the spike. The reset level for the slow conductance in the CBRD model was calculated as its value at the peak of spike-release distribution in the t*-space:

$$I_{K-Ca}:\;n(t,\Delta t_{AP})=n(t,t^{*p})$$ (13)

where t*^p^ is such that $\rho (t,t^{*p})H(t,t^{*p})=\underset{0<t^{*}<+\infty }{\max}\rho (t,t^{*})H(t,t^{*})$.

### Parameters

$${\bar{g}}_{DR}^{E}={\bar{g}}_{DR}^{I}=4\;\mu S/{\mathrm{cm}}^{2},$$

$${\bar{g}}_{M}^{E}=0.7\;\mu S/{\mathrm{cm}}^{2},\;{\bar{g}}_{M}^{I}=0,$$

$${\bar{g}}_{K-Ca}^{E}=0.4\;\mu S/{\mathrm{cm}}^{2},\;{\bar{g}}_{K-Ca}^{I}=0,$$

$$\tau_{m}^{0,E}=C/g_{\mathrm{tot}}^{0,E}=28\;\mathrm{ms},\;\tau_{m}^{0,I}=C/g_{\mathrm{tot}}^{0,I}=9\;\mathrm{ms},$$

$$V_{th,0}^{E}=-50\;\mathrm{mV},\;V_{th,0}^{I}=-45\;\mathrm{mV},$$

$$V_{\mathrm{reset}}=-40\;\mathrm{mV},\;\Delta t_{AP}=1.5\;\mathrm{ms},$$

$$C=1\;\mu F/{\mathrm{cm}}^{2},\;\sigma_{V}=3(1+g_{syn}/g_{tot}^{0})mV,$$

$$S=3\cdot 10^{-5}\;{\mathrm{cm}}^{2}$$

$$V_{L}=-70mV.$$

Here $g_{tot}^{0}$ is the total conductance at rest, and *g*_*syn*_ is the total synaptic conductance; *S* is the membrane area; *σ*_*V*_ is the noise amplitude meaning the dispersion of individual neuron’s voltage fluctuations in a stationary state. Its scaling with *g*_*syn*_ approximately reflects the synaptic noise increase with the increase of mean synaptic drive [72].

The noise term *I*_*noise*_ in Eq 2 is nonzero only for the interneurons (Fig 11). It is calculated as an Ornstein-Uhlenbeck process with the amplitude 25pA and the correlation time 4ms.

The depolarisation block is modeled through the dynamic threshold as $V_{th}^{E,I}=V_{th,0}^{E,I}+100mV\;Sigmoid(U_{\infty }-V_{DB})/7mV),$ where *Sigmoid*(*x*) = *1*/(*1*+exp(-*x*)), *V*_*DB*_ = −40*mV*, $\frac{dU_{\infty }}{dt}=\frac{U(t,t*)-U_{\infty }}{100ms}$.

When calculating the dynamics of a neural population, the integration of Eqs 2–9 determines the evolution of the distribution of voltage *U*^*E*^ across t*. Then, the effect of crossing the threshold and the diffusion due to noise are taken into account by *H*-function, Eq 4, substituted into the equation for neuronal density, Eq 1. The integral Eq 10 results in the output firing rate *ν*^*E*^(*t*).

### Connections

The synaptic conductances are described with the second-order differential equations [73] with introduced synaptic plasticity factors $x_{glu}^{D}(t)$ and $x_{GABA}^{D}(t)$, i.e. as follows

$$g_{AMPA,j}(t)={\bar{g}}_{AMPA,j}m_{AMPA,j}(t)x_{glu}^{D}(t),$$ (14)

$$g_{NMDA,j}(t,U^{j})={\bar{g}}_{NMDA,j}\;f_{NMDA}(U^{j}(t))m_{NMDA,j}(t)x_{glu}^{D}(t),$$ (15)

$$f_{NMDA}(V)=1/(1+Mg/3.57\exp(-0.062V)),$$

$$g_{GABA,j}(t)={\bar{g}}_{GABA,j}\;m_{GABA,j}(t)x_{GABA}^{D}(t),$$ (16)

forj=EandI

where *Mg* is the magnesium (Mg^2+^) concentration in mM; *m*_*s*,*j*_(*t*) is the non-dimensional synaptic conductance which is approximated by the second-order ordinary differential equation:

$$(\tau_{r}^{s,j}\tau_{d}^{s,j}\frac{d^{2}}{dt^{2}}+(\tau_{r}^{s,j}+\tau_{d}^{s,j})\frac{d}{dt}+1)m_{s,j}(t)=\tau^{s,j}(1-m_{s,j}(t))\varphi_{i}(t),$$ (17)

$$\tau^{s,j}=(\tau_{r}^{s,j}-\tau_{d}^{s,j})/({\left(\tau_{d}^{s,j}/\tau_{r}^{s,j}\right)}^{\tau_{d}^{s,j}/(\tau_{r}^{s,j}-\tau_{d}^{s,j})}-{\left(\tau_{d}^{s,j}/\tau_{r}^{s,j}\right)}^{\tau_{r}^{s,j}/(\tau_{r}^{s,j}-\tau_{d}^{s,j})}),$$ (18)

$$if\;\tau_{r}^{s,j}\neq \tau_{d}^{s,j},$$

$$\tau_{r}^{s,j}e,\;otherwise.$$

Here *φ*_*i*_ is the presynaptic firing rate. In neglect of spatial propagation and temporal delays, the presynaptic firing rate is equivalent to the somatic firing rate, i.e. *φ*_*i*_≡*ν*_*i*_. The index *s* is the synapse type, *s* = *AMPA*, *GABA* or *NMDA*; the index *i* = *E* for *s* = *AMPA* or *NMDA* and *i* = *I* for *s* = *GABA*; *w*_*glu*_ and *w*_*GABA*_ are the synaptic weights that change because of short-term plasticity; ${\bar{g}}_{s,j}$ is the maximum conductance, $\tau_{r}^{s,j}$ and $\tau_{d}^{s,j}$ are the rise and decay time constants. We imply that the synaptic time constants are estimated from the somatic responses to the stimulation of a presynaptic neuronal population. Thus these time constants characterize not only synaptic channel kinetics but the dendritic and axonal propagation delays as well. The time scale *τ*^*s*,*j*^ is chosen in the form of Eq 21 in order to provide independence of the maximum of *g*_*s*,*j*_(*t*) on $\tau_{r}^{s,j}$ and $\tau_{d}^{s,j}$, when *g*_*s*,*j*_(*t*) is evoked by a short pulse of *φ*_*j*_(*t*).

The parameter values were as follows:

${\bar{g}}_{NMDA,E}=0.6\;mS/{cm}^{2}$, ${\bar{g}}_{NMDA,I}=0.9\;mS/{cm}^{2}$, ${\bar{g}}_{AMPA,E}=0.3\;mS/{cm}^{2}$, ${\bar{g}}_{AMPA,I}=0.4\;mS/{cm}^{2}$, ${\bar{g}}_{GABA,E}=1\;mS/{cm}^{2}$, ${\bar{g}}_{GABA,I,I}=2\;mS/{cm}^{2}$. *V*_*AMPA*_ = *V*_*NMDA*_ = 0, *Mg* = 0.25 *mM*, $\tau_{r}^{AMPA,E}=\tau_{r}^{AMPA,I}=1.7$ ms, $\tau_{d}^{AMPA,E}=\tau_{d}^{AMPA,I}=8.3$ ms, $\tau_{r}^{NMDA,E}=\tau_{r}^{NMDA,I}=6.7$ ms, $\tau_{d}^{NMDA,E}=\tau_{d}^{NMDA,I}=100$ ms, $\tau_{r}^{GABA}=0.5$ ms, $\tau_{d}^{GABA}=20$ ms. The GABA reversal potential is determined by the ionic dynamics.

### Short-term synaptic plasticity

The synaptic depression was modeled with the Tsodyks-Markram model [74]:

$$\frac{dx_{glu}^{D}}{dt}=\frac{\left(1-x_{glu}^{D}\right)}{\tau_{glu}}-U_{glu}\;x_{glu}^{D}\;\varphi_{E}(t),$$ (19)

$$\frac{dx_{GABA}^{D}}{dt}=\frac{\left(1-x_{GABA}^{D}\right)}{\tau_{GABA}}-u_{GABA}\;x_{GABA}^{D}\;\varphi_{I}(t),$$ (20)

with *τ*_*glu*_ = *τ*_*GABA*_ = 500 ms, *u*_*glu*_ = 0.2, and *u*_*GABA*_ = 0.1.

A crucial role of short-term synaptic depression in discharge termination was found in experiments and modeling [75]. Simulations with the absence of synaptic depression show that the neuronal populations spontaneously switch to a high-activity state without termination.

### Representative neurons

Representative neurons of each of the populations were modeled by the basic single neuron model with the same synaptic inputs as for the populations. This model is described by the equations for the membrane voltage, Eqs 2,3,5–13, where the sum of partial derivatives was substituted by the total derivative in time *t*, and the sodium current was explicitly present in the right-hand part of Eq 2. The sodium current dependent on voltage *V* was approximated as in [71]:

$$I_{Na}(t)={\bar{g}}_{Na}\;m^{3}(t)h(t)(V(t)-V_{Na}),$$ (21)

$$\frac{dm}{dt}=\alpha_{m}(U)(1-m)-\beta_{m}(U)m,$$ (22)

$$\frac{dh}{dt}=\alpha_{h}(U)(1-h)-\beta_{h}(U)h,$$ (23)

$$\alpha_{m}=-0.32(U+50)/(\exp(-(U+50)/4)-1),$$

$$\beta_{m}=0.28(U+23)/(\exp(-(U+23)/5)-1),$$

$$\alpha_{h}=0.128\;\exp(-(U+46)/18),$$

$$\beta_{h}=4/(1+\exp(-(U+23)/5)),$$

with ${\bar{g}}_{Na}=7\;\mu S/cm^{2}$ and *V*_*Na*_ = 50*mV*.

### Ionic dynamics

**Fig 12:**
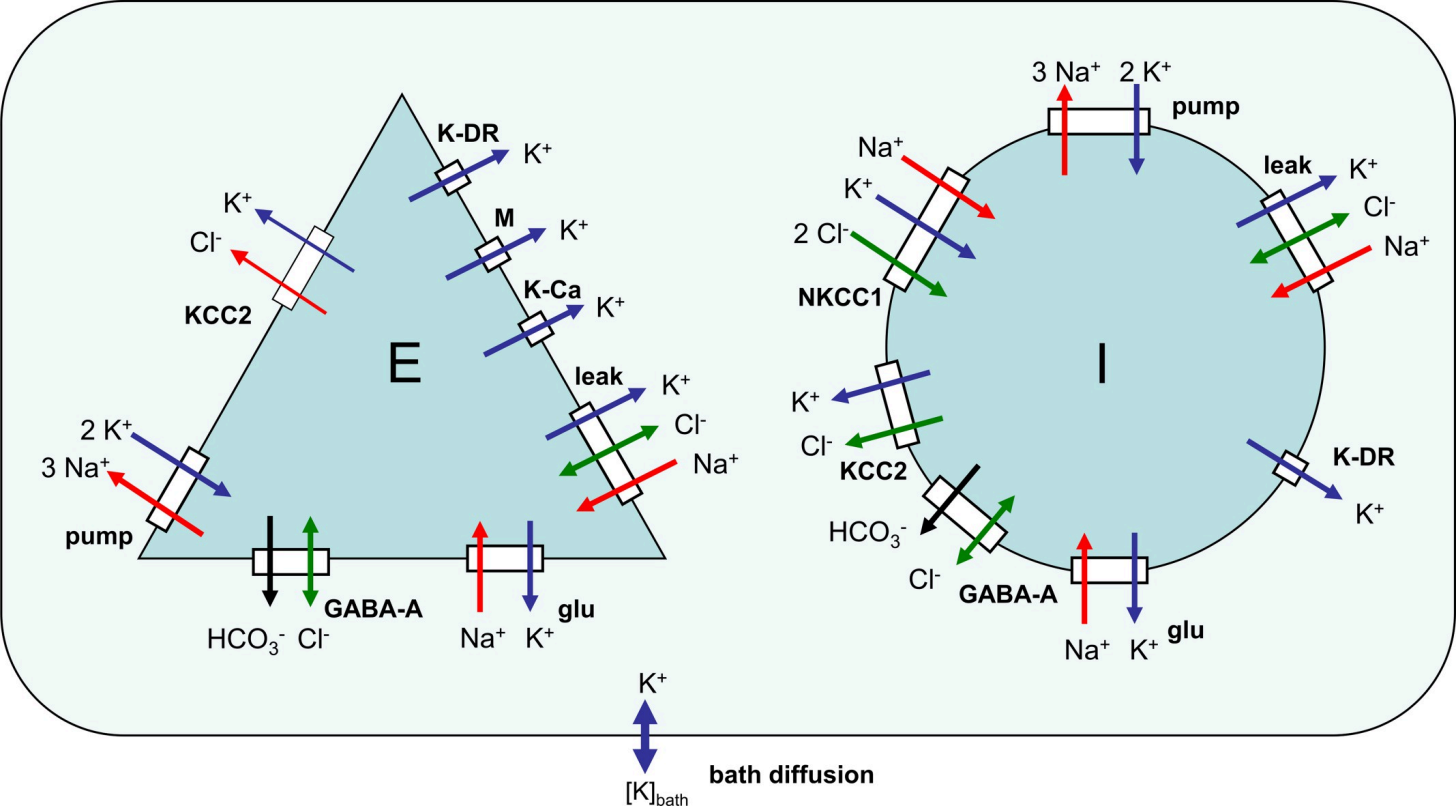
A schematic diagram of ionic dynamics mechanisms. *E* and *I* are the populations of pyramidal cells and interneurons. The dynamics of K^+^, Cl^–^, Na^+^, and HCO_3_^–^ ions as determined by the action of the leak, voltage-gated K-DR, A, M, AHP, synaptic GABA_A_, and glutamatergic channels together with the K^+^/Cl^−^cotransporters KCC2 and NKCC1 and the Na^+^/K^+^ exchange pump.

The ionic concentrations that strongly affect reversal potentials are the extracellular potassium concentration [*K*^+^]_*o*_, the intracellular chloride ion concentrations ${\left[Cl^{-}\right]}_{i}^{E}$ and ${\left[Cl^{-}\right]}_{i}^{I}$ for *E-* and *I*-neurons, respectively, and the intracellular sodium concentrations ${\left[Na^{+}\right]}_{i}^{E}$ and ${\left[Na^{+}\right]}_{i}^{I}$ (Fig 12).

The potassium balance equation was modified after Wei et al. [76,77]. The contribution of inhibitory neurons *α*_*I*_ was equal to 1/4 in accordance with the fraction of inhibitory neurons in the cortical tissue. For each of the populations, *E* and *I*, the intracellular concentrations of the potassium, chloride, and sodium ions were calculated according to the balance equations:

$$\frac{d{\left[K^{+}\right]}_{i}}{dt}=\gamma [-I_{K,leak}^{}-I_{K,active}^{}-I_{K,glu}^{}+2I_{pump}^{}+I_{KCC2}^{}+I_{NKCC1}^{}]$$ (24)

$$\frac{d{\left[Cl^{-}\right]}_{i}}{dt}=\gamma (I_{Cl,leak}+I_{GABA}+I_{KCC2}+2I_{NKCC1})$$ (25)

$$\frac{d{\left[Na^{+}\right]}_{i}}{dt}=\gamma (-I_{Na,leak}-I_{Na,glu}+q_{Na}\nu (t)-3I_{pump}+I_{NKCC1}+I_{NCX})$$ (26)

$$\frac{d(v{\left[K^{+}\right]}_{o})}{dt}=-\beta [(1-\alpha_{I})\frac{d{\left[K^{+}\right]}_{i}^{E}}{dt}+\alpha_{I}\frac{d{\left[K^{+}\right]}_{i}^{I}}{dt}]+G+D_{bath}({\left[K^{+}\right]}_{bath}-{\left[K^{+}\right]}_{o})+D_{1d}(\frac{\partial^{2}{\left[K^{+}\right]}_{o}}{\partial x^{2}})$$ (27)

$$\frac{d(v{\left[Cl^{-}\right]}_{o})}{dt}=-\beta [(1-\alpha_{I})\frac{d{\left[Cl^{-}\right]}_{i}^{E}}{dt}+\alpha_{I}\frac{d{\left[Cl^{-}\right]}_{i}^{I}}{dt}]$$ (28)

$$\frac{d(v{\left[Na^{+}\right]}_{o})}{dt}=-\beta [(1-\alpha_{I})\frac{d{\left[Na^{+}\right]}_{i}^{E}}{dt}+\alpha_{I}\frac{d{\left[Na^{+}\right]}_{i}^{I}}{dt}]$$ (29)

where *β* is the ratio of intra/extracellular volume; $I_{K,leak}^{E/I}$ and $I_{K,active}^{E/I}$ are the potassium currents through the leak and active voltage-gated channels; $I_{K,glu}^{E/I}$ is the potassium current through glutamatergic channels; $I_{pump}^{E/I}$ is the current of potassium dublets, provided by the Na^+^/K^+^ pump; $I_{KCC\;2}^{E/I}$ is the flux of potassium due to the KCC2-cotransporter; $I_{NKCC1}^{I}$ is the potassium flux due to the NKCC1-cotransporter taken into account only for the *I*-population; *γ* is the surface-to-volume and charge-to-concentration translating parameter; *I*_*Cl*,*leak*_ and *I*_*Na*,*leak*_ are the chloride and sodium ion leak currents, respectively; *I*_*GABA*_ is the chloride ion current through GABA-A-controlled receptors; and *I*_*Na*,*glu*_ is the sodium current through the glutamatergic receptors. Reversal potentials were obtained from the Nernst equations:

$$V_{K}=26.6mV\;\ln({\left[K^{+}\right]}_{o}/{\left[K^{+}\right]}_{i})$$

$$V_{Cl}=26.6mV\;\ln({\left[Cl^{-}\right]}_{i}/{\left[Cl^{-}\right]}_{o})$$

$$V_{Na}=26.6mV\;\ln({\left[Na^{+}\right]}_{o}/{\left[Na^{+}\right]}_{i})$$

$$V_{GABA}=26.6mV\;\ln(\left(4{\left[Cl^{-}\right]}_{i}+{\left[HCO_{3}^{-}\right]}_{i})/(4{\left[Cl^{-}\right]}_{o}+{\left[HCO_{3}^{-}\right]}_{o}\right))$$

The KCC2 [78] and NKCC1 [77,79] transporter currents were calculated as follows:

$$I_{pump}=\frac{I_{pump,\max}}{\left(1+\exp(3.5-{\left[K^{+}\right]}_{o}))(1+\exp((25-{\left[Na^{+}\right]}_{i})/3)\right)}$$

$$I_{KCC\;2}=I_{KCC\;2,\max}(V_{K}-V_{Cl})/(V_{K}-V_{Cl}-40\;mV)$$

$$I_{NKCC1}=I_{NKCC1,\max}(V_{Na}+V_{K}-2V_{Cl})/26.6\;mV$$

The Na^+^/K^+^ pump was taken from [80]:

$$I_{pump}(U,{\left[K^{+}\right]}_{i},{\left[K^{+}\right]}_{o},{\left[{Na}^{+}\right]}_{i},{\left[{Na}^{+}\right]}_{o})=I_{pump,max}\frac{\alpha_{1}^{+}\alpha_{2}^{+}\alpha_{3}^{+}\alpha_{4}^{+}+\alpha_{1}^{-}\alpha_{2}^{-}\alpha_{3}^{-}\alpha_{4}^{-}}{16.8S_{\alpha \beta }},$$

where $S_{\alpha \beta }={\alpha_{1}^{+}\alpha_{2}^{+}\alpha_{3}^{+}+\alpha }_{1}^{+}\alpha_{2}^{+}\alpha_{4}^{+}+\alpha_{3}^{+}\alpha_{2}^{+}\alpha_{4}^{+}+\alpha_{2}^{+}\alpha_{3}^{+}\alpha_{4}^{+}+\alpha_{1}^{-}\alpha_{3}^{-}\alpha_{4}^{-}+\alpha_{1}^{-}\alpha_{2}^{-}\alpha_{4}^{-}+\alpha_{2}^{-}\alpha_{3}^{-}\alpha_{4}^{-}+\alpha_{1}^{-}\alpha_{2}^{-}\alpha_{3}^{-}+\alpha_{4}^{+}\alpha_{2}^{-}\alpha_{1}^{-}+\alpha_{4}^{+}\alpha_{2}^{-}\alpha_{1}^{+}+\alpha_{4}^{-}\alpha_{2}^{+}\alpha_{3}^{+}+\alpha_{4}^{-}\alpha_{2}^{+}\alpha_{3}^{-}+\alpha_{1}^{+}\alpha_{2}^{+}\alpha_{3}^{-}+\alpha_{1}^{+}\alpha_{2}^{-}\alpha_{3}^{-}+\alpha_{1}^{-}\alpha_{4}^{-}\alpha_{3}^{+}+\alpha_{1}^{-}\alpha_{3}^{+}\alpha_{4}^{+}$; $\alpha_{1}^{-}=8.605$, $\alpha_{2}^{-}=\frac{40\;{\tilde{{Na}_{e}}}^{3}}{{\left(1+\tilde{{Na}_{e}}\right)}^{3}+{\left(1+\tilde{K_{e}}\right)}^{2}-1}$, $\alpha_{3}^{-}=\frac{79300{\left[P\right]}_{i}[H^{+}]}{1+\tilde{MgATP}},\;\alpha_{4}^{-}=\frac{40\;{\tilde{K_{i}}}^{2}}{{\left(1+\tilde{{Na}_{i}}\right)}^{3}+{\left(1+\tilde{K_{i}}\right)}^{2}-1}$, $\alpha_{1}^{+}=\frac{1050\;{\tilde{{Na}_{i}}}^{3}}{{\left(1+\tilde{{Na}_{i}}\right)}^{3}+{\left(1+\tilde{K_{i}}\right)}^{2}-1}$, $\alpha_{2}^{+}=481$, $\alpha_{3}^{+}=2000\frac{{\tilde{K_{e}}}^{2}}{{\left(1+\tilde{{Na}_{e}}\right)}^{3}+{\left(1+\tilde{K_{e}}\right)}^{2}-1}$, $\alpha_{4}^{+}=\frac{320\;\tilde{MgATP}}{\tilde{1+MgATP}}$ with $\tilde{{Na}_{i}}=\frac{{\left[{Na}^{+}\right]}_{i}}{2.49\;exp(-0.386U)},\;\tilde{{Na}_{e}}=\frac{{\left[{Na}^{+}\right]}_{o}}{15.5\;exp(12.1U)},\;\tilde{K_{i}}={\left[K^{+}\right]}_{i}/0.5$, $\tilde{K_{e}}={\left[K^{+}\right]}_{o}/0.213,\;[H^{+}]=1e-4,\tilde{MgATP}=3.904,\;{\left[P\right]}_{i}=4.2/(1+{\left[K^{+}\right]}_{i}/292+[H^{+}]/6.77+{\left[{Na}^{+}\right]}_{i}/224)$, where the concentrations are in mM, the voltage U is in volts here.

Ionic channel currents were calculated based on the membrane potentials averaged across the entire population:

$$\bar{U}(t)=\int_{0}^{\infty }U(t,t^{*})\rho (t,t^{*})dt^{*}$$

The leak currents are as follows:

$$I_{K,leak}=g_{KL}(\bar{U}(t)-V_{K}(t)),\;I_{Cl,leak}=g_{ClL}(\bar{U}(t)-V_{Cl}(t)),\;I_{Na,leak}=g_{NaL}(\bar{U}(t)-V_{Na}(t)),$$

$$I_{GABA}^{}=g_{GABA}^{}(t)(\bar{U}(t)-V_{GABA}^{}(t)),$$

$$I_{K,active}=\int_{0}^{\infty }(I_{K-DR}(t,t^{*})+I_{M}(t,t^{*})+I_{K-Ca}(t,t^{*}))\rho (t,t^{*})dt^{*}$$

is the average potassium current through the voltage-gated channels. The M- and K-Ca-components are absent for interneurons for simplicity.

The potassium and sodium currents through the glutamatergic channels [81] were approximated as linear dependent on voltage with the fractions of the total glutamatergic conductance. The fraction was estimated as 0.2 for the potassium and 0.4 for the sodium ions [82]:

$$I_{K,glu}^{}=0.2g_{glu}^{}(\bar{U}-V_{K}),\;I_{Na,glu}^{}=0.4g_{glu}^{}(\bar{U}-V_{Na}).$$

The glial buffer is [40]:

$$G=k_{1}(B_{\max}-B)/k_{1N}-k_{2}B,$$ (30)

$$\frac{dB}{dt}=k_{1}(B_{\max}-B)-k_{2}B,\;k_{2}=k_{1}/(1+\exp(-({\left[K^{+}\right]}_{o}-15mM)/1.15mM)).$$

The ***parameters*** are as follows:

$\gamma =S/(F\;vol)=10^{-3}\;[(mM/ms)/(\mu A/cm^{2})]$; *β* = 10; *α*_*I*_ = 0.25. The Na^+^/K^+^ pump, KCC2, and NKCC1 transporter current amplitudes are as follows: *I*_*pump*,max_ = 0.8 *μA*/*cm*^2^, $I_{KCC2,\max}^{E}=I_{KCC2,\max}^{I}=1\mu A/cm^{2}$, $I_{NKCC1,\max}^{E}=0$, $I_{NKCC1,\max}^{I}=0.1\mu A/cm^{2}$, [*Cl*^−^]_*o*_ = 130 *mM*, [*K*^+^]_*i*_ = 129 *mM*, [*Na*^+^]_*o*_ = 130 *mM*, ${\left[HCO_{3}^{-}\right]}_{o}=24mM$, and ${\left[HCO_{3}^{-}\right]}_{i}=16mM$. The leak conductances are the same for both populations: *g*_*KL*_ = 20 *μS*/*cm*^2^, *g*_*CIL*_ = 20 *μS*/*cm*^2^, and *g*_*NaL*_ = 7 *μS*/*cm*^2^. The membrane area is 3∙10^−5^*cm*^2^. The sodium charge transferred by a single spike is *q*_*Na*_ = 0.1 *μC*/*cm*^2^. The potassium concentration mixture with the bath solution was characterized with the parameter *D*_*bath*_ = 0.25*s*^−1^. The coefficient of the spatial diffusion of potassium ions is *D*_1*d*_ = 0.39 *μm*^2^/*ms*. The initial concentrations are [*K*^+^]_*o*_ = [*K*^+^]_*bath*_ = 3.5*mM*, ${\left[Cl^{-}\right]}_{i}^{E}=5\;mM$, ${\left[Cl^{-}\right]}_{i}^{I}=9\;mM$, and ${\left[Na^{+}\right]}_{i}^{E}={\left[Na^{+}\right]}_{i}^{I}=17\;mM$. The glial pump was neglected in all simulations except the one focused on its effect, where *k*_1_ = 0.02 *s*^−1^, *B*_*max*_ = 500 *mM*.

The calcium concentration inside neurons increases mainly due to the influx through the NMDA receptors and decreases due to the outflow through the sodium-calcium exchangers. The effect of the exchanger is approximated as a relaxation term $I_{NCX}^{E}={\left[Ca^{2+}\right]}_{i}^{E}/\left(\gamma \tau_{Ca}\right)$ in the equation for the calcium concentration inside *E*-neurons

$$\frac{d{\left[Ca^{2+}\right]}_{i}^{E}}{dt}=\frac{-{\left[Ca^{2+}\right]}_{i}^{E}}{\tau_{Ca}}+\chi g_{NMDA,E}(t)(U^{E}(t)-V_{Ca}),$$ (31)

where the time constant of the relaxation due to Na^+^/Ca^2+^ exchanger *τ*_*Ca*_ = 200 *ms*, the contribution of the calcium ions into glutamatergic ionic transport *χ* = 0.001 and the reversal potential *V*_*Ca*_ = 20*mV*. The calcium dynamics was neglected for interneurons.

### Volume equation

Extracellular space (ECS) volume dynamics has been described phenomenologically, as in [19]:

$$\frac{dv}{dt}=\frac{1}{\tau_{v}}(1+0.1029\beta^{0}(\exp(\frac{\Delta \pi }{20})-1)-v),$$ (32)

where $v=v_{o}/v_{o}^{0}$ is the ratio of the ECS volume to its initial value; *β*^0^ = 10 is the initial ratio of intracellular versus ECS volumes; *τ*_*v*_ = 250 *ms* is the characteristic time constant of the volume change because of the change of osmolarity Δ*π*: $\Delta \pi ={\left[Na^{+}\right]}_{i}+{\left[Cl^{-}\right]}_{i}+{\left[K^{+}\right]}_{i}-{\left[Na^{+}\right]}_{o}-{\left[Cl^{-}\right]}_{o}-{\left[K^{+}\right]}_{o}-\Delta \pi^{0}$, where $\Delta \pi^{0}={\left[Na^{+}\right]}_{o}^{0}+{\left[Cl^{-}\right]}_{o}^{0}+{\left[K^{+}\right]}_{o}^{0}+{\left[HCO_{3}^{-}\right]}_{o}^{}-{\left[Na^{+}\right]}_{i}^{0}-{\left[Cl^{-}\right]}_{i}^{0}-{\left[K^{+}\right]}_{i}^{0}-{\left[HCO_{3}^{-}\right]}_{i}^{}$.

### Equation of neuronal activity propagation

The horizontal cortical connections are supposed to be local and isotropic. They are determined by the relationship between the somatic rates *v*^*i*^ and presynaptic firing rates *φ*_*i*,*j*_, where *i* and *j* are the indexes of the pre- and postsynaptic populations, respectively. In the case of 1-d geometry, all variables depend on the spatial coordinate *x*, oriented along with the layers of the cortical tissue. A Gaussian profile of the strengths of the connections is assumed, i.e.

$$\varphi_{i,j}(t,x)=\int \nu^{i}(t,x')e^{-{\left(x-x'\right)}^{2}/\lambda_{}^{2}}dx'/\int e^{-{\left(x-x'\right)}^{2}/\lambda_{}^{2}}dx'$$ (33)

where *λ* is the characteristic length, assumed to be equal for all types of connections, *λ* = 50 *μm*, which roughly corresponds to electrophysiological estimations from paired recordings [83]. In 1-d representation, the cortical area was considered as a segment of the length 2.5mm. The spatial inhomogeneity of parameters was set with the linear distribution of ${\bar{g}}_{AMPA,E}$, which was equal to zero at *x* = 0, and maximum at *x* = *L*.

### Software and source code

The model was implemented in the software “Brain” that is available from the public repository with DOI: https://doi.org/10.6084/m9.figshare.15113370.v1.

### Estimation of activity pattern characteristics

To obtain statistical data from simulations, we varied realizations of the noise term *I*_*noise*_ in Eq 2. From 5 realizations of 400s simulations, we used the second, third and fouth IDs, thus obtaining n = 15 values for each characteristic. We estimated the following characteristics of the activity: the speed of the wavefronts, the interdischarge frequency, and the maximum value of the extracellular potassium ion concentration reached during IDs. The first IDs after the onset of the simulation were excluded. For each ID, the speed was estimated from the slope of the slowest fragment of the wavefront, as shown with the black lines in Fig 4B–4D. Similar values were obtained in estimations of a time lag between the K+ transients in remote sites at half the peak amplitudes as indicated in Fig 7A; in this case, the ID speed was calculated as a ratio of the distance between recorded neurons and the time lag. The ID frequency was measured as the reverse time between the fronts at one location S1, so as the maximum extracellular potassium ion concentration. The results are expressed as the median and 25%-75% interquartile range.
